# Alkali Counterion-Dependent Crystallization of Uranium(IV)–Chloro Structural Units

**DOI:** 10.1021/acs.inorgchem.5c00822

**Published:** 2025-06-12

**Authors:** Madeline C. Shore, Jennifer N. Wacker, Pere Miró, Jeffery A. Bertke, Karah E. Knope

**Affiliations:** † Department of Chemistry, 8368 Georgetown University, Washington, D.C. 20057, United States; ‡ Department of Chemistry, University of Iowa, Iowa City, Iowa 52242, United States

## Abstract

The synthesis, structural characterization, and spectroscopic properties of five tetravalent uranium (U) phases including Li_6_[U_4_(μ_3_-O)_2_Cl_18_(H_2_O)_2_]·10H_2_O (**1**), [U­(H_2_O)_4_Cl_4_] (**2**), [U­(H_2_O)_4_Cl_4_]·KCl (**3**), Rb_2_UCl_6_ (**4**), and Cs_2_UCl_6_ (**5**) are reported. Notably, a change in the U^4+^ solid-state structural unit was observed based on the identity of the alkali counterion used in the synthesis. Li^1+^ yielded a tetranuclear oxo-bridged cluster, [U_4_(μ_3_-O)_2_Cl_18_(H_2_O)_2_]^6–^, Na^1+^ and K^1+^ yielded two structurally distinct [U­(H_2_O)_4_Cl_4_] complexes, and Rb^1+^ and Cs^1+^ resulted in [UCl_6_]^2–^ as the dominant phases. The spectroscopic properties of the compounds were analyzed using Raman and UV–vis–NIR absorption spectroscopy. The UV–vis–NIR spectra of compounds **1**–**5** exhibited transitions consistent with uranium in the +4 oxidation state. Clear differences in the absorption band splitting were observed and are likely attributed to differences in metal ion coordination, crystal field effects, and outer sphere interactions Overall, this work demonstrates the utility of noncovalent interactions in tuning the crystallization of various metal complexes from otherwise identical reaction solutions and provides further evidence that counterions impact the composition and structure of actinide complexes isolated in the solid state. In this way, this work affords important insight into directing and controlling the structure of actinide complexes and clusters.

## Graphical abstract

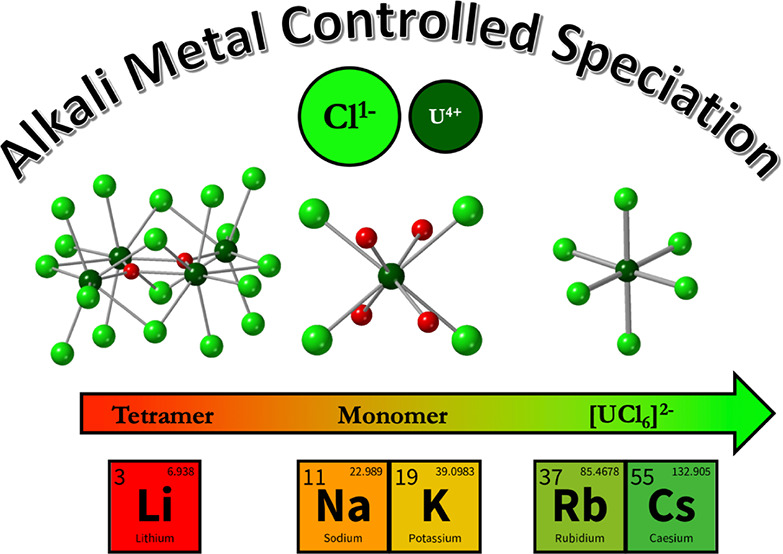

## Introduction

Outer sphere counterions are ubiquitous in inorganic chemistry as charge balancing species, but can also significantly influence the coordination environment and behavior of metal ions.^1–8^ Toward this end, counterions have been leveraged to modulate catalysis,^9–12^ increase compound stability,^13–15^ and understand structure–property relationships of inorganic compounds.^16–18^ To highlight one example, noncovalent interactions (NCIs) have been shown to impact the properties of single molecule magnets, with intramolecular hydrogen bonding stabilizing ligand coordination, and thereby affecting the magnetic behavior of the complexes.^19,20^ Despite extensive evidence of the importance of NCIs in inorganic chemistry, their impact on the chemical and physical properties of *f*-elements remains largely unexplored. This knowledge gap limits our ability to predict and control actinide chemistry under chemically complex conditions such as those found in nuclear waste management,^21,22^ or dynamic biological environments present in radiomedicine.^23,24^ Therefore, advancing our understanding of outer coordination sphere ions and their influence on the structure, stability, and reactivity of *f*-element compounds remains a critical need.

Several studies highlight the importance of NCIs in *f*-element chemistry.^25–33^ Specifically, NCIs have been shown to influence solid-state structural chemistry, redox behavior, and vibrational and optical properties.^34–38^ For example, examination of lanthanide metal ions in molten chloride salts showed that countercation identity impacted the redox potential of Eu^3+^.^39^ For the actinides, Jin et al. demonstrated that tetra-*n*-alkylammonium cations help stabilize the higher oxidation states of neptunium under aqueous conditions.^40^ However, directly probing these interactions remains challenging. Crystallization, together with complementary analytical techniques, affords one approach for elucidating the effects of NCIs. For instance, Forbes et al. utilized structural analysis, thermochemistry, and Raman spectroscopy to demonstrate that NCIs weaken the Np=O bond.^41^

With this in mind, our group and others have been exploring the effects of alkali metal identity on actinide solid-state structural chemistry. Past work has shown that alkali counter-cations can stabilize clusters of varying nuclearity, affect solubility, and drive speciation.^27,42,43^ For example, Soderholm et al. examined the effects of countercation hydration enthalpies on thorium-nitrate complexation,^42^ and showed a correlation between hydration enthalpy and thorium complex composition and charge. Additionally, Sigmon and Hixon demonstrated that outer sphere alkali metal cations can promote the isolation of actinide-oxo clusters.^43^ Moreover, Lin et al. examined the solvothermal syntheses of U^4+^ sulfate complexes, and found that smaller cations such as Na^1+^ yield hexameric [U_6_O_4_(OH)_4_(H_2_O)_4_(SO_4_)_12_]^12–^ clusters, while Cs^1+^ precipitates trimeric [U_3_O-(SO_4_)_7_]^4–^ units.^44^ Such findings point to alkali countercation-controlled U^4+^ speciation and highlight the need to further investigate the effects of NCIs on the structural chemistry of U^4+^-complexes from different ligand systems.

In this work, the synthesis and solid-state structural chemistry of U-chloride complexes prepared in the presence of various alkali metal cations were examined. Five compounds were synthesized, and structural analysis by single crystal X-ray diffraction revealed the isolation of three unique structural units: [U_4_(μ_3_-O)_2_Cl_18_(H_2_O)_2_]^6–^, [U­(H_2_O)_4_Cl_4_], and [UCl_6_]^2–^. Whereas the more charge-dense metal (i.e., Li^1+^) yielded polynuclear units as the major phase, the less charge-dense metals (i.e., Rb^1+^ and Cs^1+^) tended to precipitate [UCl_6_]^2–^ motifs as the dominant products. The crystallization behavior of the complexes further underscores how counterion identity can be utilized as a strategy to selectively precipitate unique U-chloride structural units from aqueous solution. Given the relevance of halide media to various aspects of actinide chemistry, including spent fuel processing and modern nuclear energy technologies, such observations are highly significant and critical to our understanding of actinide behavior.

## Experimental Section

Caution! ^238^U (*t*_1/2_ = 4.5 × 10^9^ years) is an α-emitting radionuclide. As such, standard precautions for handling radioactive materials should be followed when performing the syntheses described.

### Materials

Uranium tetrachloride, UCl_4_, was synthesized following the procedure reported by Kiplinger et al.^45^ Chemicals used for the preparation of UCl_4_ included UO_3_ (International BioAnalytical Industries, Inc.), hexachloropropene (Alfa Aesar), and dichloromethane (Fisher Chemicals). Additionally, the following chemicals were used as received from commercial suppliers: lithium chloride (LiCl; Sigma-Aldrich), sodium chloride (NaCl; Fisher Scientific), potassium chloride (KCl; Fisher Scientific), rubidium chloride (RbCl; Sigma-Aldrich), cesium chloride (CsCl; Fisher Scientific), lithium hydroxide (LiOH; Fisher Scientific), sodium hydroxide (NaOH; Sigma-Aldrich), potassium hydroxide (KOH; Sigma-Aldrich), ammonium hydroxide (NH_4_OH, Sigma-Aldrich). Concentrated hydrochloric acid (HCl, Sigma-Aldrich) was diluted into nanopure water (≤0.05 μS); water was purified by a Millipore Direct-Q 3UV water purification system.

### Syntheses

Five compounds including Li_6_[U_4_(μ_3_-O)_2_Cl_18_(H_2_O)_2_]·10H_2_O (**1**), [U­(H_2_O)_4_Cl_4_] (**2**), [U­(H_2_O)_4_Cl_4_]·KCl (**3**), [Rb_2_UCl_6_] (**4**), and [Cs_2_UCl_6_] (**5**), were prepared following a general synthetic procedure that involved solvent evaporation of a U^4+^/HCl aqueous solution that contained alkali counterions.

A solution was prepared by dissolving UCl_4_ (0.108 g, 0.284 mmol) in water (1 mL). An aliquot of concentrated NH_4_OH (300 μL) was then added and a green, amorphous solid precipitated. The mixture was centrifuged for 5 min at 4500 rpm in a 50 mL falcon tube; the supernatant was then discarded. The resulting precipitate (nominally U­(OH)_4_) was washed with 1 mL of water (3X) and then dissolved in 1 mL of 4 M HCl. Aliquots from this acidic U_(aq)_ stock solution (100 μL, 0.026 mmol U, 6.26 mg U) were pulled and combined with 400 μL 4 M HCl. Based on literature precedence, dissolution of the precipitate in HCl presumably yields a U­(H_2_O)_*x*_Cl_*y*_ complex.^46^ To each solution, aliquots of 1 M ACl_(aq)_ A = Li, Na, K, Rb, Cs (50 μL, 0.05 mmol) were added. The reaction solvent was allowed to evaporate under a nitrogen atmosphere at room temperature. After approximately 4–8 days, green crystals were observed. Note that while this general synthetic procedure yielded crystals of **1**–**5**, variation of the ACl/AOH ratio was found to produce better quality crystals for **2** and **3**; nevertheless, PXRD analysis showed that under both conditions, the major phase matched well with the calculated diffraction patterns of compounds **2** and **3**, respectively. Details of these syntheses are provided in the Supporting Information.

### Single Crystal X-ray Diffraction

The structures for compounds **1**–**4** were determined using single crystal X-ray diffraction. A full data set was not collected for **5** as the structure was previously reported by Schleid and Morss et al.^47^ Data were collected on a Bruker Quest D8 diffractometer equipped with a IμS X-ray source (Mo Kα radiation; λ = 0.71073 Å) and a CMOS detector. Single crystals were isolated from the bulk reaction products and mounted in mineral oil on MiTeGen micromounts. Data were collected at 100 K. The APEX III software suite was used to identify unit cells, integrate the data, and apply absorption corrections.^48^ The structures were solved using intrinsic phasing methods and refined using *SHELXL* within the *ShelXle* graphical user interface.^49^ Crystallographic refinement details for **1**–**4** are provided in Table 1. Further refinement details are available as Supporting Information.

**Table 1:** Crystallographic Refinement Details for Compounds **1**–**4**

	**1**	**2**	**3**	**4**
formula	Cl_18_H_12_Li_5_O_14_U_4_	Cl_4_H_8_O_4_U	Cl_5_H_8_KO_4_U	Cl_6_Rb_2_U
MW (g mol^–1^)	1861.02	451.89	526.44	621.67
T (K)	100	100	100	100
crystal color/habit	green blade	green block	green prism	green shard
crystal system	monoclinic	monoclinic	monoclinic	trigonal
space group	*C* 2*/m*	*C* 2*/c*	*C* 2*/c*	*P*31*c*
λ (Å)	0.71073	0.71073	0.71073	0.71073
*a* (Å)	19.2652(9)	13.4899(19)	14.1554(6)	7.4057(3)
*b* (Å)	9.0976(5)	6.5994(9)	13.4705(6)	7.4057(3)
*c* (Å)	11.4076(6)	11.3715(16)	12.9007(6)	11.5742(7)
α (deg)	90	90	90	90
β (deg)	110.403(2)	119.366(4)	107.532(2)	90
γ (deg)	90	90	90	120
Volume (Å^3^)	1873.94(17)	882.3(2)	2345.64(18)	549.74(6)
Z	2	4	8	2
ρ (mg m^–3^)	3.298	3.402	2.981	3.756
μ (mm^–1^)	18.557	19.561	15.305	24.946
*R*_1_	0.0284	0.0377	0.0176	0.0217
*wR*_2_	0.0732	0.0964	0.0423	0.0460
GOF	1.083	1.095	1.110	1.144
CCDC	2407423	2407422	2407424	2407421

### Powder X-ray Diffraction (PXRD)

Powder X-ray diffraction patterns for the reaction products that yielded single crystals of **1**–**5** were collected to confirm that the single crystals used for structure determination were representative of the bulk (Figures S9–S17). Data were collected on ground samples on a Bruker D8 Quest equipped with a Photon III detector using a Mo IμS source. A series of 360° phi scans were obtained with the sample to detector distance set to 150 mm. Data were integrated in the range of 3 to 38° 2θ.

### Vibrational Spectroscopy

Raman spectra were collected on single crystals of **1**–**5** (Figures S18–S22) at room temperature on a Horiba LabRAM HR Evolution Raman Spectrometer with an excitation line of 532 nm over Δυ 200–2000 cm^–1^ using circularly polarized radiation.

### UV–Vis–NIR Absorption Spectroscopy

UV–vis-NIR absorption spectra were collected on single crystals of **1**–**5** (Figures S23–S33). Crystals were placed on a microscope slide and data were collected at room temperature from 200 to 1000 nm on a CRAIC Technologies model 508 PV microspectrophotometer mounted on a Zeiss Axioscope 5.

## Results and Discussion

Five tetravalent uranium chloride bearing phases were isolated from acidic aqueous chloride solutions using different alkali chloride salts. The compounds were synthesized via solvent evaporation of a U^4+^/HCl aqueous solution and subsequently characterized using structural analysis and spectroscopic techniques. The structures are comprised from three unique building units, including a tetranuclear [U_4_(μ_3_-O)_2_Cl_18_(H_2_O)_2_]^6–^ cluster, and monomeric [U­(H_2_O)_4_Cl_4_] and [UCl_6_]^2–^ molecular complexes. Notably the composition and structure of the dominant phase in each reaction product showed dependence on the alkali counterion used in the synthesis. A summary of the compounds isolated including descriptions of the cation used in the synthesis, whether or not it incorporated into the crystallized phases, the nuclearity of the complex, the U site symmetry, and supramolecular network dimensionality are provided in Table S9.

### Structure Descriptions and Structural Systematics

Single crystals of compound **1**, Li_6_[U_4_(μ_3_-O)_2_Cl_18_(H_2_O)_2_]·10H_2_O, suitable for structure determination were isolated using Li^1+^. Interestingly, powder X-ray diffraction (PXRD) patterns for the Na^1+^ and Rb^1+^ reaction products exhibit peaks consistent with the calculated pattern for **1**, suggesting that an isomorphous phase can be isolated with these ions (Figures S12, S16). However, these compounds were only present as minor phases (approximately 5–10%), and single crystals could not be obtained for structural analysis. As such, only the Li^1+^ analog is described. Compound **1**, Li_6_[U_4_(μ_3_-O)_2_Cl_18_(H_2_O)_2_]·10H_2_O, crystallizes in the *C*2/*m* space group. As shown in Figure 1a, four U metal centers are bridged through μ_2_-Cl and μ_3_-O groups to form a tetranuclear cluster core of composition [U_4_(μ_3_-O)_2_Cl_6_]^6+^. Eighteen chlorides and two water molecules terminate the cluster to form the structural unit, [U_4_(μ_3_-O)_2_Cl_18_(H_2_O)_2_]^6+^. U1 is eight-coordinate, bound to three monodentate chlorides, three μ_2_-Cl, and two μ_3_-O groups. U2 is similarly eight coordinate, bound to three monodentate chlorides, three μ_2_-Cl, one μ_3_-O, and one water molecule. Average U-μ_3_-O, U-μ_2_-Cl, U–Cl, and U–H_2_O distances are 2.23(2), 2.83(3), 2.70(2), and 2.50(6) Å, respectively. Distances between adjacent U are 3.813(2)–4.043(2) Å for U1---U2 and 3.709(2) Å for U1---U1i. The U2---U2i distance is 6.928(3) Å. Six lithium cations as well as ten water molecules exist in the outer coordination sphere (Figure 1b); average Li---Cl and Li---O_(H2O)_ distances range from 2.5853(2) Å to 2.8512(3) Å and 1.9076(3) Å to 2.0973(3) Å, respectively. Lattice water molecules interact with Cl of the U cluster via H-bonding interactions; the shortest O–H_(H2O)_---Cl distance is 3.228(5) Å with an O–H---Cl angle of 138(7)°.^50^ Hydrogen bonding also exists between outer sphere water molecules, with an O–H_(H2O)_---O_(H2O)_ interaction distance and angle of 2.875(11) Å, 100(6)°.^51^ Taken together, these noncovalent interactions yield an overall 3D supramolecular network (Figure S5).

**Figure 1:**
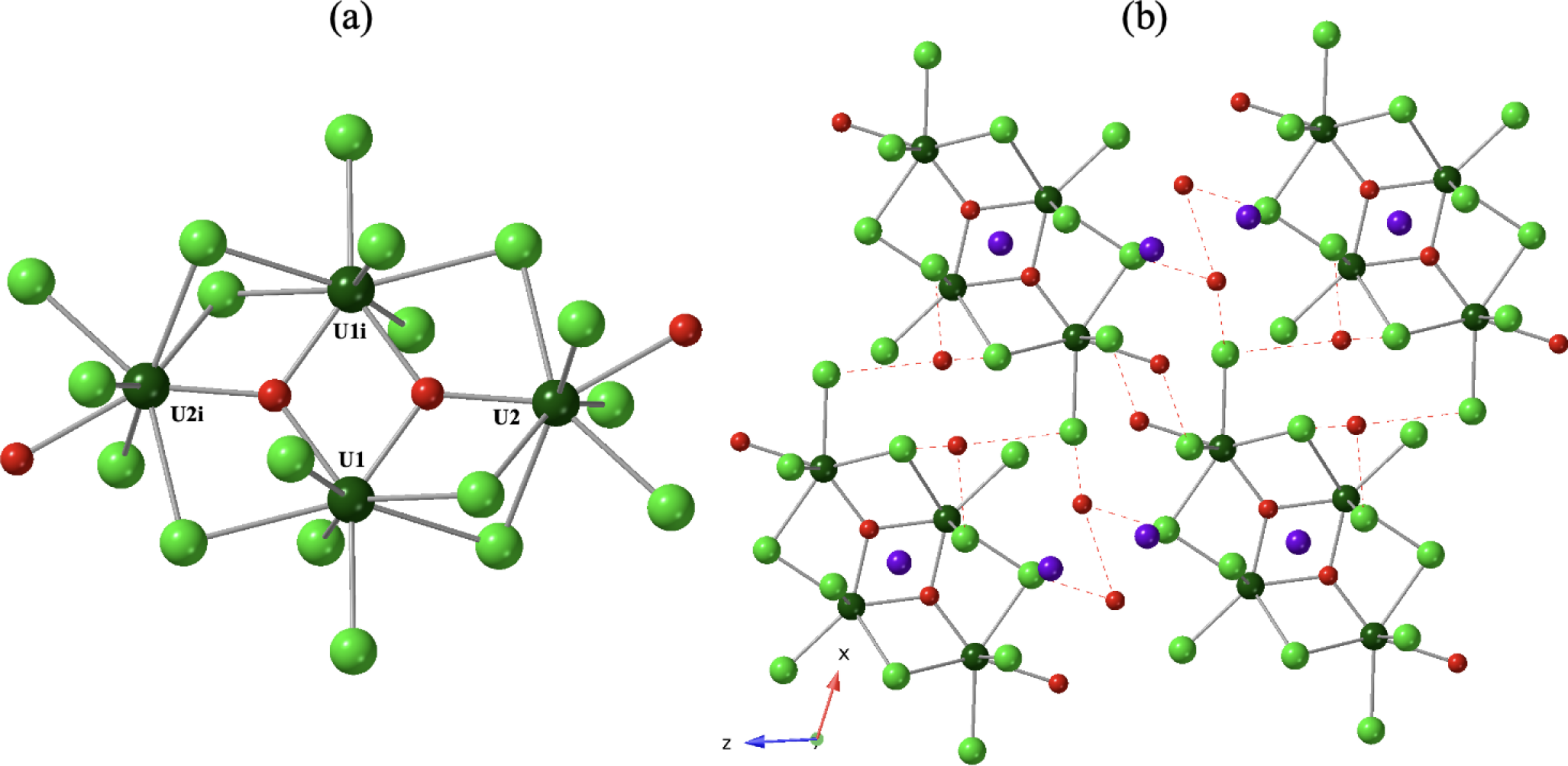
(a) Ball and stick representation of the [U_4_(μ_3_-O)_2_Cl_18_(H_2_O)_2_]^6–^ cluster in **1**. (b) Packing diagram of Li_6_[U_4_(μ_3_-O)_2_Cl_18_(H_2_O)_2_]·10H_2_O viewed down the [010]. Color code: U, dark green; O, red; Cl, green; Li, purple. Hydrogen atoms are omitted for clarity. The symmetry operator 1 – x, y, 1 – z is denoted by i. Red dashed lines indicate hydrogen bonding interactions.

Relatively few homometallic tetramers have been isolated for U^4+^. A search of the Cambridge Structural Database (version 2024.3.0) for U^4+^ tetrameric units with metal centers bridged by Cl,^52^ N,^53–55^ and/or O yielded 25 hits.^56^ Of these, ten are μ_2_-, μ_3_-, or μ_4_-oxo bridged and adopt one of three known tetrameric units. These include the planar [U_4_O_2_]^12+^ core found in **1**, a μ_2_-oxo bridged [U_4_O_4_]^8+^ motif,^53,57^ and a relatively rare μ_4_-oxo bridged [U_4_O]^14+^ cluster.^58^ Interestingly, nearly all of these structures have been prepared under nonaqueous conditions. Four of the reported U tetramers adopt a [U_4_O_2_] core.^59,60^ The previously reported, [U_4_O_2_(bdc)_3_(form)_7_], shares the same μ_3_-O bridged, planar, tetrameric unit as **1**, but is terminated by terephthalate (bdc) and formate (form) ligands.^61^ By comparison, [[U_4_Cl_2_O_2_(L^2^)_2_(H_2_O)­(THF)]·3­(THF)][Bibr ref62] (where L^2^ is p-tert butylhexahydroxy[2.1.2.1.2.1]­metacyclophane) similarly consists of the [U_4_O_2_]^12+^ moiety with the U^4+^ metal centers additionally bridged by μ_2_-Cl groups. The structure of [U_4_Cl_10_O_2_(THF)_6_(2-FA)_2_]·2THF is the closest structural analog of **1**.^63^ This compound consists of the same tetranuclear cluster, with the U metal centers bridged by μ_2_-Cl ligands and bound to terminal chlorides. However, the tetramer is also terminated by 2-furoate (FA) and THF ligands, which differentiate it from **1**. Moreover, **1** was isolated under entirely aqueous conditions unlike the phases previously reported.

Compound **2,** [U­(H_2_O)_4_Cl_4_], crystallizes in the *C*2/*c* space group and was isolated using Na^1+^ and K^1+^ (Figure S12), though neither cation incorporated into the crystal structure. The U^4+^ metal center is eight coordinate, bound to four chlorides and four water molecules forming a neutral [U­(H_2_O)_4_Cl_4_] complex. U–Cl and U–O­(H_2_) distances range from 2.689(2)–2.695(2) Å and 2.421(6)–2.442(6) Å, respectively. As shown in Figure 2, strong hydrogen bonding interactions exist between the bound water molecules and chlorides of adjacent structural units, with O–H_(H2O)_---Cl distances and angles ranging from 3.086(7) Å, 174(10)° to 3.270(8) Å, 163(9)°. Overall, the H-bonding interactions connect the [U­(H_2_O)_4_Cl_4_] structural units into a 3-dimensional, supramolecular network (Figure S6).

**Figure 2:**
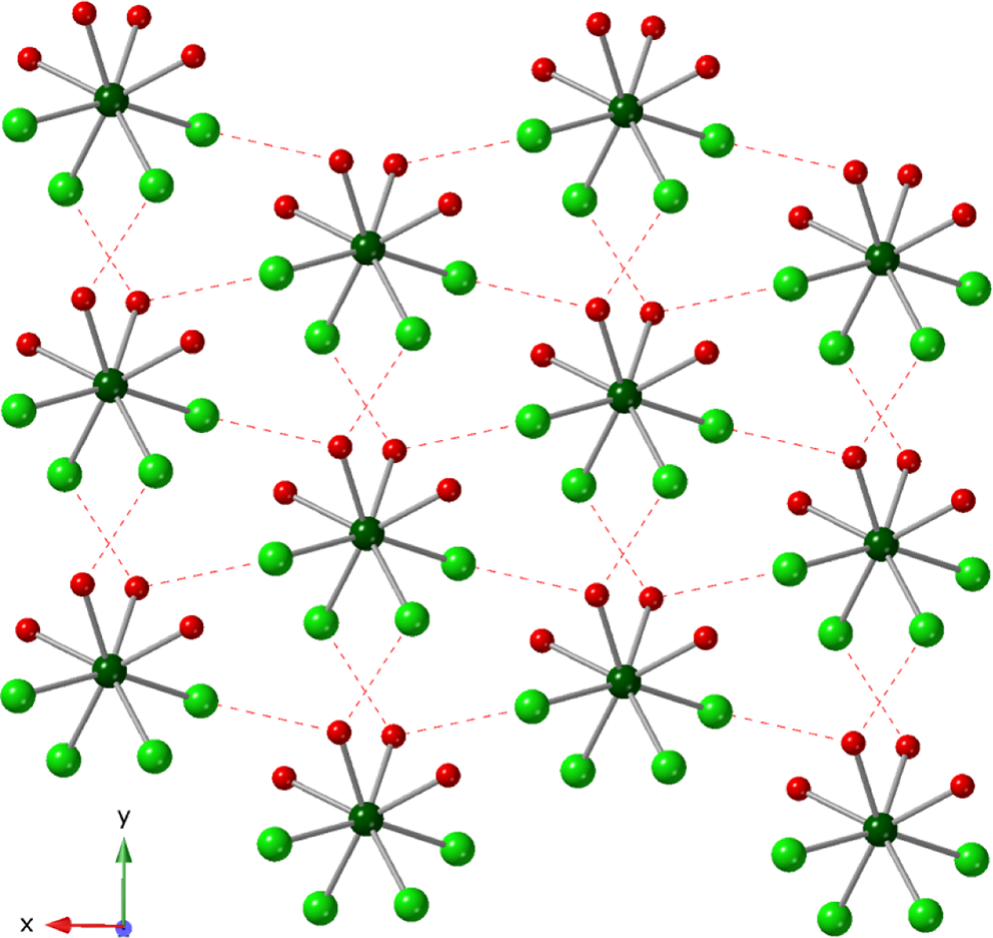
Packing diagram of compound **2**, [U­(H_2_O)_4_Cl_4_], viewed down the [001]. Eight coordinate U are shown as dark green spheres. Oxygen is shown in red and Cl in light green. Hydrogen bonding interactions are shown as red dashed lines. Hydrogen atoms have been omitted for clarity.

Compound **3,** [U­(H_2_O)_4_Cl_4_]·KCl, crystallizes in the *C*2/*c* space group. Based on powder X-ray diffraction data (Figure S14) this phase was isolated using Na^1+^ and K^1+^; however, single crystals suitable for structure determination were only prepared with K^1+^. As such, the K^1+^ analog is described. Like the complex in **2**, the U center is bound to four chlorides and four water molecules forming a charge neutral [U­(H_2_O)_4_Cl_4_] structural unit (Figure 3). Bond distances for U–Cl and U–O­(H_2_) range from 2.653(9)–2.720(9) Å and 2.439(3)–2.414(2) Å, respectively. Relatively strong hydrogen bonding interactions exist between coordinated water molecules and chloride ions of adjacent [U­(H_2_O)_4_Cl_4_] complexes; O_(H2O)_---Cl_(bound)_ distances and angles range from 3.149(3) Å, 167(3)° to 3.361(3) Å, 160(3)°. Potassium and chloride ions exist in the outer coordination sphere. Water molecules of U complexes interact with lattice Cl with O_(H2O)_---Cl_(lattice)_ distances and angles ranging from 3.048(3) Å, 164(4)° to 3.167(3) Å, 156(3)°. K---Cl interaction distances range from 3.052(2) to 3.160(3) Å. As shown in Figure S7, the O–H---Cl interactions link U­(H_2_O)_4_Cl_4_ units into an overall 3D supramolecular network.

**Figure 3:**
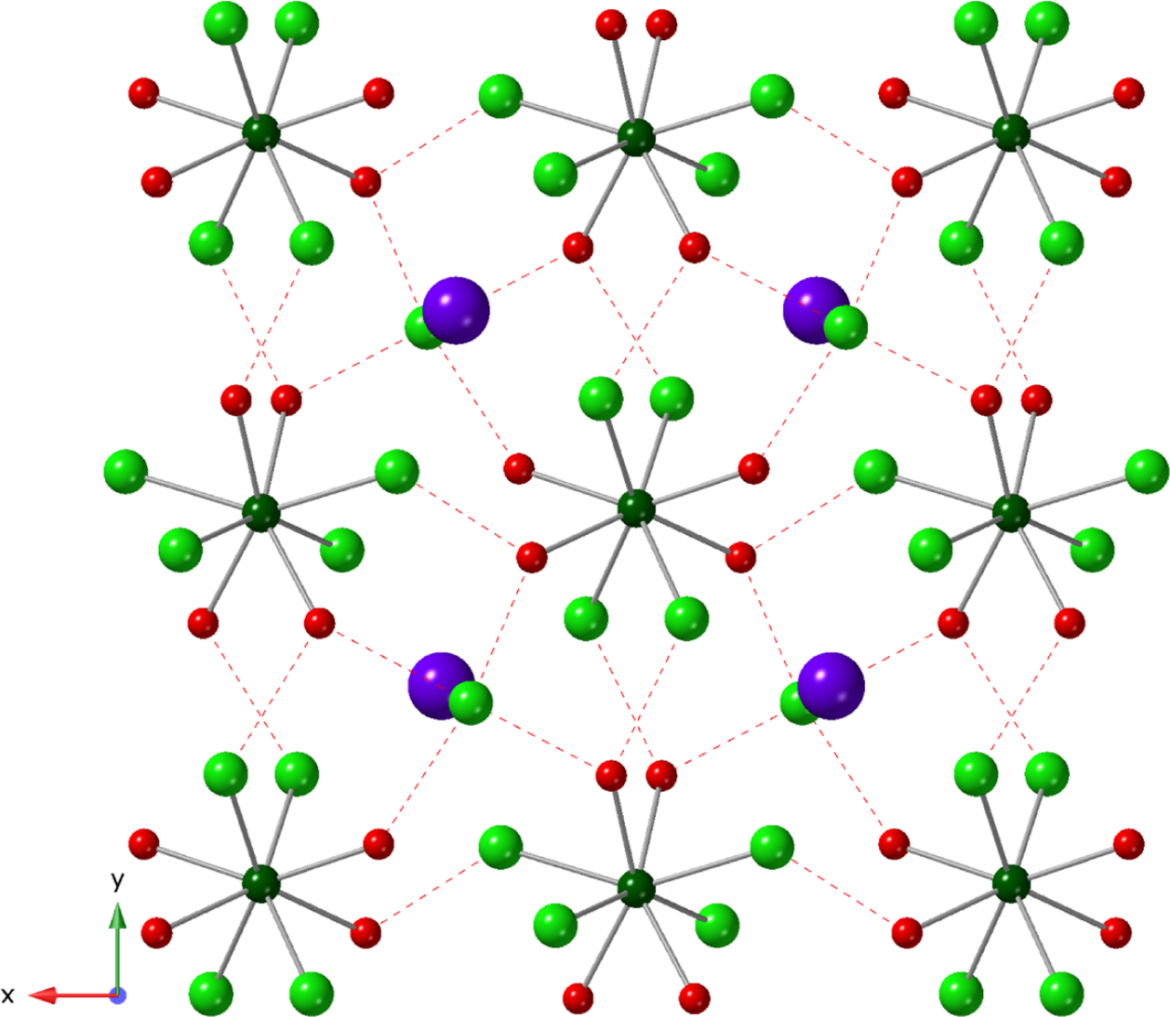
Packing diagram of [U­(H_2_O)_4_Cl_4_]·KCl (**3**) viewed down the [001]. Dark green atoms are 8-coordinate U metal centers. Oxygen, chloride, and potassium are shown in red, light green, and purple, respectively. Hydrogen bonding interactions are shown as red dashed lines. Hydrogen atoms have been omitted for clarity.

The complexes in the structures of **2** and **3**, as well as a [U­(H_2_O)_4_Cl_4_] unit previously isolated using HN-heterocycles,^30,64^ are isomers and exhibit differences in the distribution of chloride and water molecules about the U metal center. Shown in Figure 4 are the two U­(H_2_O)_4_Cl_4_ complexes isolated in this work as well as the U­(H_2_O)_4_Cl_4_ complex previously reported by Wacker et al.^64^ The previously described unit (Figure 4a) consists of a U metal center bound to a plane of four chlorides with one water molecule above and three water molecules below the plane. By comparison, the complex in **2** (Figure 4b) is composed of a belt of three chlorides and one water molecule, with one chloride above and three water molecules below the plane. The complex in **3** (Figure 4c) adopts the same plane as **2**, consisting of three chlorides and one water molecule; however, the U is bound to one water molecule above, and one chloride and two water molecules below the belt. Notably two of the U structural units (a,c) depicted in Figure 4 are isomorphic with Th–Cl–H_2_O complexes previously reported by our group.^38^ For the Th^4+^ complexes, ESP calculations showed that the partitioning of Cl^1–^ and H_2_O about the Th centers was largely dependent on the p*K*_a_ of the NH heterocycles used in the synthesis. Further, variation in the arrangement of Cl^1–^ and H_2_O about the metal center resulted in differences in the polarizability of the complexes.

**Figure 4:**
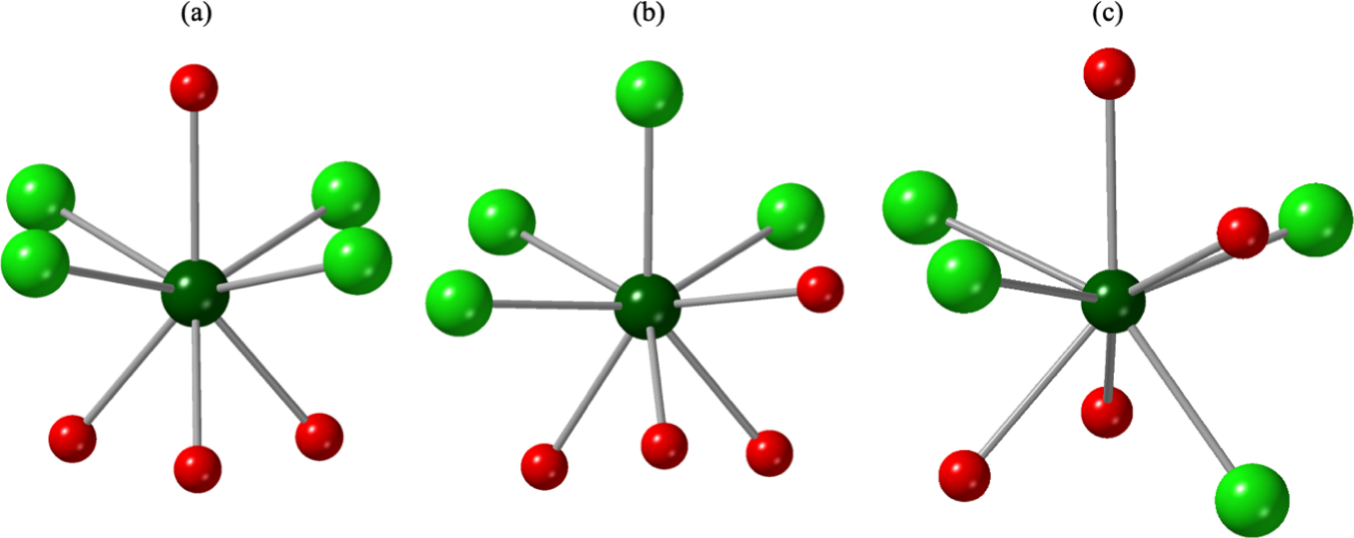
Ball and stick representation of the [U­(H_2_O)_4_Cl_4_] complexes (a) previously reported by Wacker et al.,^64^ (b) in **2**, and (c) in **3**.

Compound **4,** Rb_2_UCl_6_, crystallizes in the *P*31*c* space group. The uranium center adopts an octahedral coordination geometry with *C*_3_ site symmetry. As shown in Figure 5, the U is bound to six chlorides forming an overall anionic [UCl_6_]^2–^ structural unit. U–Cl bond distances range from 2.617(3)–2.628(2) Å. Rubidium ions exist in the outer coordination sphere and exhibit Rb---Cl interaction distances that range from 3.3728(3)–3.6390(2) Å.

**Figure 5:**
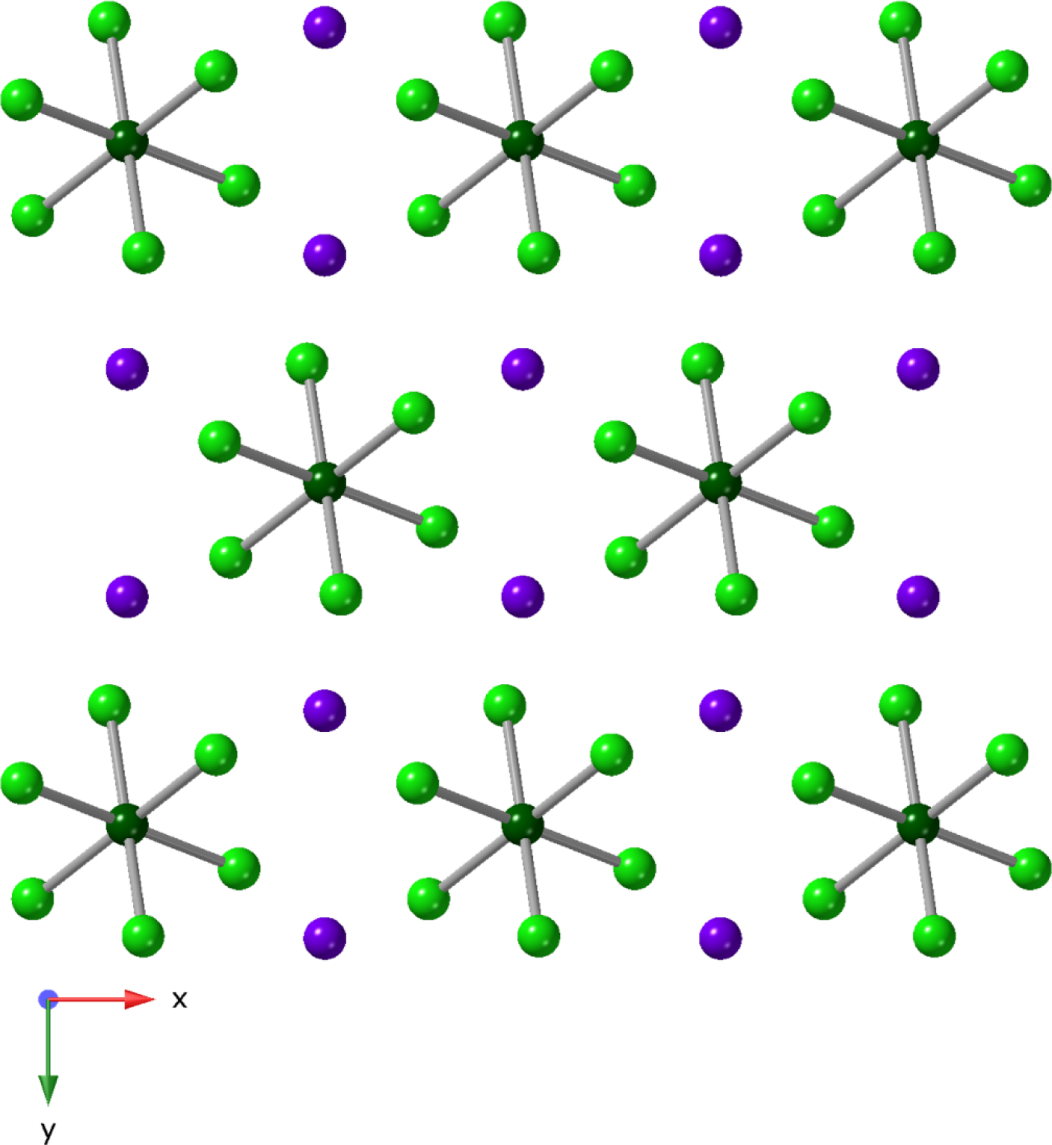
Packing diagram of **4**, Rb_2_UCl_6_, viewed down the [001]. Color code: U, dark green; Cl, green; Rb, purple.

Compound **5**, Cs_2_UCl_6_, was previously reported by Schleid and Morss.^47^ However, a brief description is given here as it relates to the current work. Compound **5,** like **4,** is built from [UCl_6_]^2–^ structural units with the U bound to six chlorides. In contrast to **4**, the U has *O*_*h*_ site symmetry. U–Cl bond distances reported by Schleid and Morss are 2.615(2) Å and Cs---Cl distances range from 3.7562(2)–3.7679(3) Å. Notably their data was collected at 298 K.

Actinide hexachloride dianionic [AnCl_6_]^2–^ complexes are reported for Th, U, Np, and Pu.^65–68^ Such structural units have been prepared as starting materials and to understand trends in bonding and reactivity.^66^ While these dianionic complexes are pervasive in the solid state, by comparison only relatively recently have An^4+^-aquo-halide complexes been crystallized using outer coordination sphere interactions.^30^ Nonetheless, U^4+^-aquo chloro complexes related to those observed in **2** and **3** have been identified in solution using X-ray Absorption Spectroscopy.^46^ Interestingly, these solution studies suggest that aquo-chloro complexes are prevalent at chloride concentrations up to 9 M. Likewise, the UV–vis-NIR absorption spectra of the reaction solutions from which **1**, **3**, **5** were isolated (Figure S33) are consistent with monomeric U–H_2_O–Cl complexes,^64^ despite the differences observed in the solid-state reaction products.

### Countercation-Dependent Precipitation of U^4+^ Structural Units

There is growing evidence that outer coordination sphere interactions impact structural chemistry, bonding, and redox behavior.^40,41,69^ In this work, three unique structural units were isolated by varying the identity of the alkali counterion used in the synthesis, with Li^1+^ yielding an oxo bridged tetramer, Na^1+^ and K^1+^ yielding mononuclear U­(H_2_O)_4_Cl_4_ complexes, and Rb^1+^ and Cs^1+^ yielding UCl_6_^2–^ units as the major phases under otherwise identical synthetic conditions. Isolation of various U complexes upon variation of alkali metal cations as observed herein is consistent with previous reports. Soderholm et al., for example, examined the influence of alkali counter cations on the solid-state structural chemistry of thorium nitrate molecular complexes.^42^ Using alkali metal cations and H^1+^, three structural units including [Th­(NO_3_)_4_(H_2_O)_3_], [Th­(NO_3_)_5_(H_2_O)_2_]^1–^, and [Th­(NO_3_)_6_]^2–^ were isolated. Interestingly, alkalis with a lower hydration enthalpy (i.e., Cs^1+^) tended to associate with less hydrated, more anionic complexes; (i.e., [Th­(NO_3_)_6_]^2–^), and those with higher hydration enthalpies (i.e Li^1+^, Na^1+^, K^1+^) yielded more hydrated complexes (i.e., [Th­(NO_3_)_5_(H_2_O)_2_]^1–^). A similar trend is observed in this work. Here, Na^1+^ and K^1+^yield the more hydrated neutral species, U­(H_2_O)_4_Cl_4_, whereas Rb^1+^ and Cs^1+^ readily crystallize the less hydrated, more anionic [UCl_6_]^2–^ units. Such results provide further evidence that counterions affect composition of metal–ligand complexes precipitated from aqueous solution.

Beyond molecular complexes, it is well documented that alkali counterions can be used to control solubility of metal-oxo clusters.^4,37,43^ For the actinides, Hixon et al., isolated a series of plutonium metal-oxo clusters using various alkali counterions.^43^ For lithium, both Li_14_(H_2_O)_n_[Pu_38_O_56_Cl_54_(H_2_O)_8_], (Li–Pu_38_) and Li_*x*_[Pu_22_O_28_(OH)_4_Cl_28_(H_2_O)_20_]­[Pu_22_O_28_(OH)_4_Cl_26_(H_2_O)_22_)]-(H_2_O)_14_ (Li–Pu_22_) nanoclusters were precipitated from halide solutions. Sodium similarly yielded a Na_*x*_[Pu_38_O_56_Cl_36_(H_2_O)_26_]­(H_2_O)_12_ (Na–Pu_38_), as well as Na_*x*_[Pu_16_O_19_(OH)_4_Cl_18_(OH)­(H_2_O)_21_]­(H_2_O)_12_ (Na–Pu_16_). Clusters including K_6_[Pu_22_O_28_(OH)_4_Cl_30_(H_2_O)_18_]­(H_2_O)_6_Cl_4_ (K–Pu_22_), and K_*x*_[Pu_16_O_19_(OH)_4_Cl_25_(H_2_O)_15_]­[Pu_16_O_19_(OH)_4_Cl_22_(H_2_O)_18_]­(H_2_O)_23_Cl_4_ (K–Pu_16_) were isolated with K^1+^. Overall larger clusters such as (Li–Pu_38_) and (Li–Pu_22_) were isolated with smaller alkalis, while smaller clusters such as (Na–Pu_16_) and (K–Pu_16_) were precipitated with Na^1+^ and K^1+^. While only one cluster, Li_6_[U_4_(μ_3_-O)_2_Cl_18_(H_2_O)_2_]·10H_2_O, was structurally characterized as part of this work, a related trend is observed here. Notably Li^1+^ yielded a tetrameric U-oxo cluster; powder X-ray diffraction suggests such clusters can also be prepared with Na^1+^ and Rb^1+^, but precipitate as minor phases (Figures S12 and S16). It is also worth noting that examination of reaction products as a function of acid concentration provided evidence that under less acidic conditions, Li^1+^ stabilized larger oligomers. For example, reactions of a nominally U­(OH)_4_ precipitate dissolved in 0.5 or 4 M HCl in the presence of counterions were prepared. Interestingly, the PXRD pattern of the 0.5 M reaction product precipitated using Li^1+^ (Figure S10) exhibits peaks at approximately 3.2° and 4° 2θ, that are absent in the 4 M HCl sample. The peaks unaccounted for by **1** are in good agreement with the calculated pattern of K–Pu_22_,^43^ suggesting the presence of larger oligomers in the bulk phase precipitated at lower acid concentrations.

Such trends in structure based on alkali identity may result from increased ion pairing from Li^1+^ to Cs^1+^ or may otherwise be understood by considering the solvation structure of the alkali ions.^4,70–72^ Nienhuis et al. recently noted that alkali metal solvation structure influences radical reaction dynamics and similar principles may extend to the coordination chemistry of actinides in chloride-rich environments, with the solvation structure imposed by alkali cations influencing uranium complexation and hydrolysis behavior.^70^ For example, weakly hydrated cations such as Cs^1+^ may create a dynamic, less ordered solvent environment that favors chloride coordination and stabilizes complexes such UCl_6_^2–^. By comparison, more strongly hydrated cations such as Na^1+^ promote structured solvent shells, increasing water availability and favoring the formation of hydrated species such as U­(H_2_O)_4_Cl_4_. Moreover, the strength and rigidity of the solvent shell for highly charge dense metals such as Li^1+^ may enhance hydration and facilitate hydrolysis by polarizing the O–H bond and thereby promoting proton transfer from coordinated water molecules. In this way, alkali cation identity may govern uranium speciation by controlling solvent structure, ion pairing, and ligand exchange dynamics.

### UV–Vis–NIR Absorption Spectroscopy

Optical absorption spectroscopy is a powerful technique for probing oxidation state, coordination chemistry, and oligomer formation of uranium compounds.^73–78^ Given clear differences in composition, coordination environment, and the nuclearity of **1**–**5**, spectra were collected to examine how structural differences manifested in the UV–vis–NIR absorption behavior of the compounds.

The UV–vis–NIR absorbance spectra of compounds **1**–**5** are shown in Figure 6 as well as Figures S23–S33. Overall the spectra are characterized by peaks consistent with U^4+^ *f*-*f* transitions.^64^ Nonetheless, there are notable differences in peak intensity and splitting of the bands observed over 500–760 nm (Figure 6). Peaks observed in this region can be tentatively assigned as the ^3^H_4_ → ^3^P_1_, ^3^H_4_ → ^3^P_0_, ^3^H_4_ → ^1^G_4_, and ^3^H_4_ → ^1^D_2_ transitions.^79,80^ Variations in the absorption spectra of compounds **1**–**5** may be attributed to differences in coordination number, site symmetry, and crystal field effects.^73,81^ The spectrum of compound **1**, an oxo-bridged tetramer with *C*_*s*_ U metal center site symmetry (Figure 6a), exhibits characteristic U^4+^ *f-f* transitions. Previous studies of other uranium μ_3_-oxo-bridged clusters, such as [U_6_O_4_(OH)_4_]^12+^ units, have suggested that peaks centered between 610–690 nm may be diagnostic for oligomers.^63^ Such a trend is not observed for **1** as there are no clear features uniquely attributed to tetrameric units.^61^ Compounds **2** and **3** both consist of [U­(H_2_O)_4_Cl_4_] units. The complexes exhibit the same composition and U site symmetry (*C*_2_), yet differences in transition energy and peak splitting are apparent (Figure 6b,c, respectively). These differences may arise from outer-sphere effects, such as hydrogen bonding, which have been shown to affect peak intensity and contribute to distinct differences in the spectral features observed over 640–700 nm.^82^ Compounds **4** and **5** are both composed of [UCl_6_]^2–^ dianionic units, and as shown in Figure 6d,e, the absorption spectra exhibit significant differences in peak splitting, shape, and intensity. Notably, compound **5** exhibits significantly enhanced peak resolution and pronounced splitting, with 11 distinct peaks over the 620 to 700 nm range; five peaks are clearly observed for compound **4** over the same range (Figure S30).

**Figure 6:**
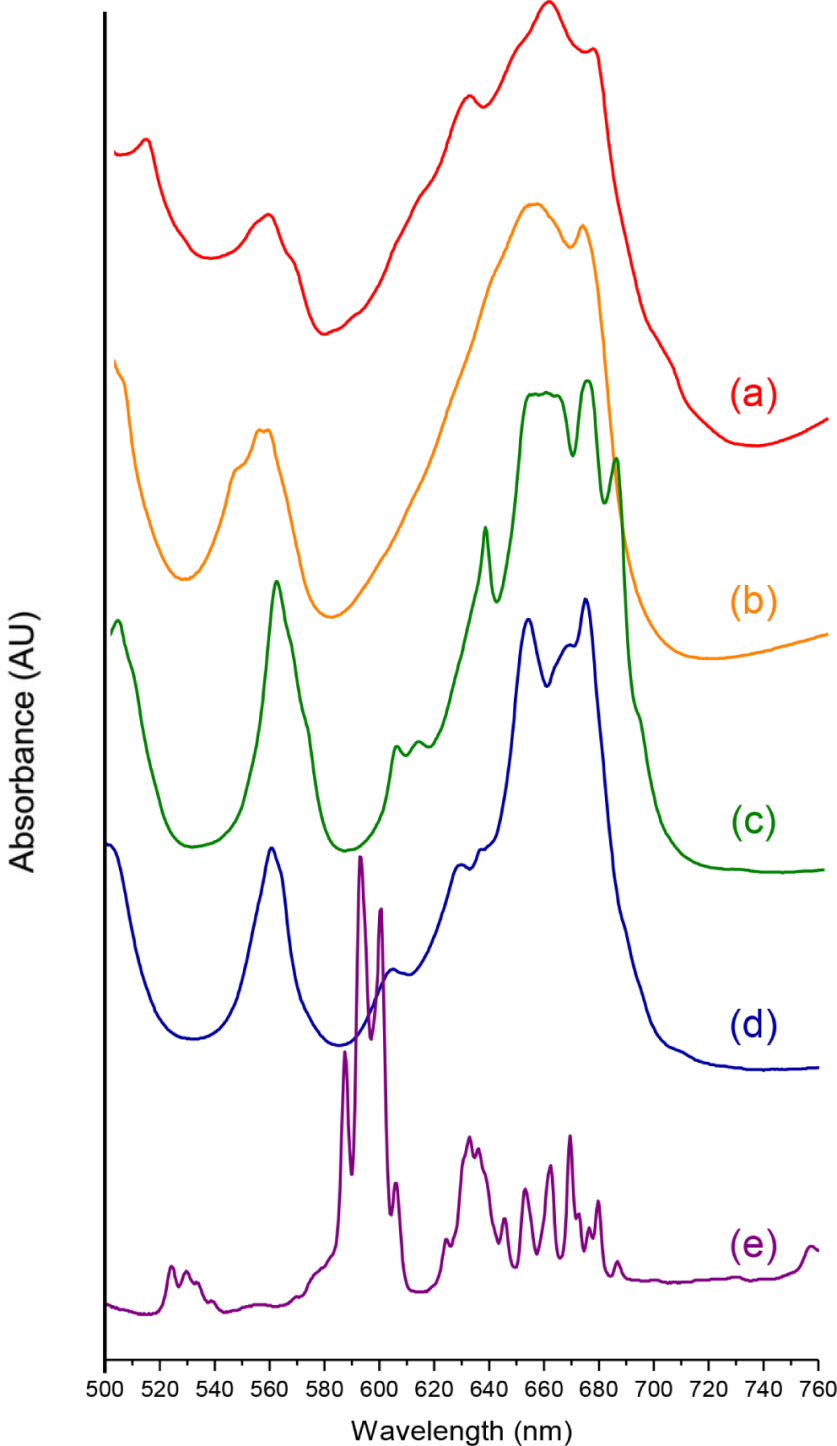
Solid-state UV–vis-NIR absorption spectra of compounds 1 (a), 2 (b), 3 (c), 4 (d), and 5 (e), plotted over 500–760 nm.

The U in **4** has *C*_3_ crystallographic site symmetry, while the metal center in compound **5** exhibits *O*_*h*_ site symmetry. These differences and resulting crystal field effects may contribute to divergent optical spectra of compounds **4** and **5**, (Figure 6d,e).^82^ Yet, it is worth noting that inspection of the coordination environments about the U metal centers in **4** and **5** show little variation in U–Cl bond distances, with **4** exhibiting U–Cl bond distances ranging from 2.617(3)–2.628(2) Å and **5** having a U–Cl distance of 2.615(2) Å. By comparison, more notable differences are observed in the interactions between the UCl_6_^2–^ anion and the outer sphere cations, Rb^1+^ and Cs^1+^, as highlighted in Figure S34. Specifically, the UCl_6_^2–^ in **4** exhibits ten close contacts with Rb^1+^ and twelve with Cs^1+^, and these differences may have a larger effect on the optical absorption behavior. This was further supported through calculations of the *f-f* absorption spectra of compounds **4** and **5** at SO-XMS-CASPT2 level of theory (Figures S35 and S36). Importantly, the calculations showed that the alkali counterions needed to be explicitly included in order to obtain good agreement between experiment and theory, pointing to importance of outer sphere interactions, and the effects of changes in packing and symmetry on properties of f-element complexes.

### Vibrational Spectroscopy

Raman spectroscopy was used to further assess the impact of coordination environment and nuclearity on vibrational properties. The spectra for **1**, **3**, and **5** are shown in Figure 7; these compounds are representative of three of the structural units reported. Metal ligand stretches are typically observed over the 200–500 cm^–1^ region.^83^ A series of peaks between 225 and 325 cm^–1^ (Figure 7) can be attributed to U–Cl and U–OH_2_ stretches.^64^ Additionally, a H_2_O bending mode is observed at 550 cm^–1^. For **1**, the peak at 425 cm^–1^ is consistent with a U-μ_3_-O vibration.^37^ Notably the spectra of **2**–**5** (Figures S18–S22) do not exhibit this peak, which may be expected as they consist of mononuclear units. In this way, this region is indicative of oligomer formation.

**Figure 7:**
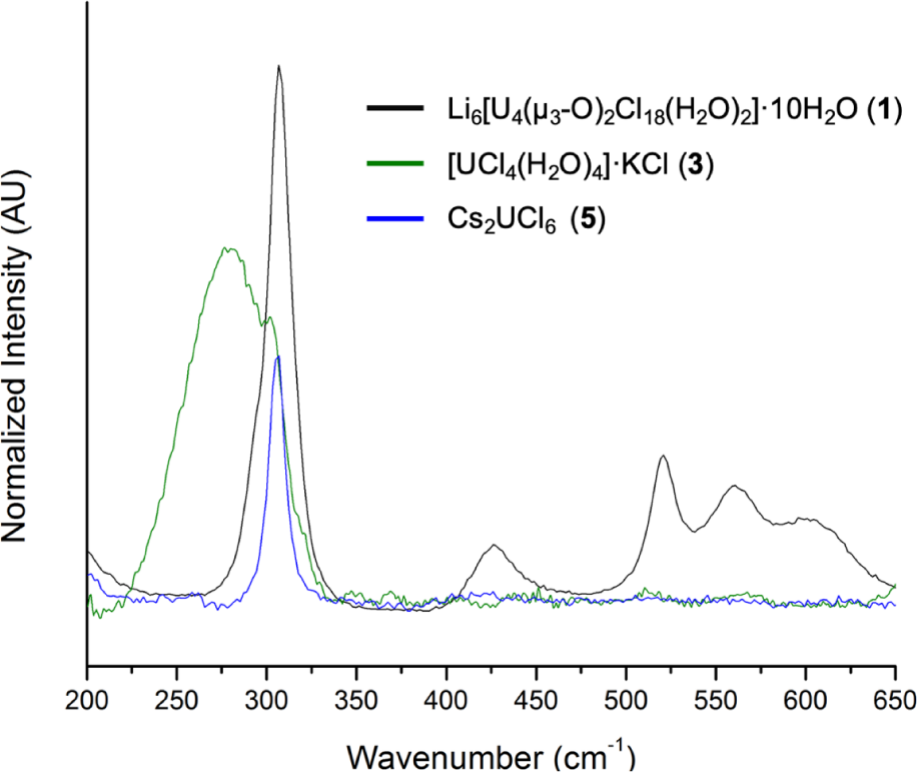
Raman spectra of **1**, **3**, and **5** plotted over 200–650 cm^–1^.

## Conclusion

Actinide structural chemistry fundamentally informs our understanding of actinide behavior, with direct implications for fields ranging from nuclear waste management to radiomedicine. In this study, five uranium-based phases were synthesized under aqueous conditions by varying the alkali metal counterions used in the reactions. Structural characterization revealed the formation of three unique structural motifs with the general formulas of [U_4_(μ_3_-O)_2_Cl_18_(H_2_O)_2_]^6–^, [U­(H_2_O)_4_Cl_4_], and [UCl_6_]^2–^. Optical absorption spectroscopy further confirmed oxidation state and highlighted the effects of crystal chemistry on the absorption behavior (i.e., peak splitting, band intensity) of the synthesized compounds, with computational studies suggesting that outer sphere interactions have a significant impact on the overall properties of actinide complexes. Examination of the synthetic parameters and structural chemistry revealed two trends: smaller alkali metal counterions tend to promote the formation of more hydrated, neutral species, and less solvent structuring ions appear to restrict cluster formation and yield less hydrated structural units. These findings are consistent with trends reported previously,^42,43^ and further highlight the influence of counterion identity on actinide structural chemistry. Such insights are critical in the context of spent fuel processing and environmental management, where such differences in speciation can impact the overall chemical behavior of uranium. The systematic trends established here extend beyond fundamental coordination chemistry and provide foundational knowledge for understanding the countercation-dependent behavior of actinide complexes under chemically complex conditions. Yet the effects of outer coordination sphere interactions on actinide structure and behavior remain insufficiently understood and warrant further investigation.

## Supplementary Material



## Notes

Additional experimental details, methods, and results including synthetic details, crystallographic refinement details, thermal ellipsoid plots, powder X-ray diffraction patterns, Raman and UV–vis-NIR spectra, summary of supramolecular interaction distances and angles, and computational details (PDF)

## §

Chemical Sciences Division, Lawrence Berkeley National Laboratory, Berkeley, California 94720, United States of America
