# Supplementary material for: Alkali Counterion-Dependent Crystallization of Uranium(IV)–Chloro Structural Units
Source: Inorg Chem. 2025 Jun 12;64(29):14799–809. doi: 10.1021/acs.inorgchem.5c00822 (PMC12308800; doi:10.1021/acs.inorgchem.5c00822)
Supplement: Supplementary file 1 [file ic5c00822_si_001.pdf]

SUPPORTING INFORMATION

ALKALI COUNTERION DEPENDENT CRYSTALLIZATION OF URANIUM(IV)-CHLORO STRUCTURAL UNITS

*Madeline C. Shore,[1] Jennifer N. Wacker,[1,2] Pere Miró,[3] Jeffery A. Bertke,[1] and Karah E. Knope[1].**

[1] Department of Chemistry, Georgetown University, Washington, D.C. 20057, United States of America
[2] Current Address: Chemical Sciences Division, Lawrence Berkeley National Laboratory, Berkeley, California 94720, United States of America
[3] Department of Chemistry, University of Iowa, Iowa City, Iowa 52242, United States
* Correspondence to kek44@georgetown.edu

Table of Contents

1. SYNTHETIC DETAILS....2
2. CRYSTALLOGRAPHIC REFINEMENT DETAILS OF COMPOUNDS 1–4....4
3. ORTEP DIAGRAMS OF COMPOUNDS 1–4....6
4. BOND VALENCE SUMMATION VALUES FOR 1....9
5. PACKING DIAGRAMS FOR 1-4....10
6. POWDER X-RAY DIFFRACTION PATTERNS FOR 1–5....14
7. RAMAN SPECTRA OF COMPOUNDS 1–5....23
8. UV-VIS ABSORPTION SPECTRA OF 1–5....28
9. SUPRAMOLECULAR INTERACTIONS OBSERVED IN COMPOUNDS 1–3....39
10. BOND DISTANCES OF COMPOUNDS 1-5....40
10. REFERENCES....45

1. SYNTHETIC DETAILS.

The influence of AOH and ACl concentrations on crystal quality was investigated. For consistency, compounds **1**-**5** were all crystallized from 4 M HCl with 50 μL of the respective chloride salt, with comparative analyses detailed below. For compounds **2** and **3**, optimized single crystals were obtained by adjusting the reagent volumes to 30 μL of ACl and 20 μL of AOH. Nevertheless, PXRD analysis showed that at both ACl/AOH ratios, the major phase matched well with the calculated diffraction patterns of compounds **2** and **3**, respectively. For structural characterization, the higher-quality single crystals were selected for collection. These studies demonstrate that, regardless of acid concentration or AOH/ACl ratio, the major crystalline phase is predominantly dictated by the identity of the alkali metal cation. However, fine-tuning both the acid concentration and AOH/ACl ratios enhances the isolation of high-quality single crystals and may also promote the formation of minor phases within the bulk product.

Synthesis of, $Li_6[U_4(\mu_3\text{-}O)_2Cl_{18}(H_2O)_2]\cdot 10H_2O$ (**1**) was synthesized via evaporation. A uranium stock solution was prepared by dissolving UCl_4 (0.108 g, 0.284 mmol) in water (1mL). An aliquot of concentrated NH_4OH (300 μL) was then added, causing a green solid to precipitate. The mixture was then centrifuged for 5 minutes at 4500 rpm in a 50 mL falcon tube and the supernatant was discarded. The resulting precipitate was washed with 1mL of water and this process was repeated until the precipitate had been washed three times. The green precipitate was then dissolved in 1 mL of 4M HCl. Aliquots from this acidic $U_{(aq)}$ stock solution (100 μL, 0.0262 mmol U, 6.26 mg U) were pulled and combined with 400 μL 4M HCl. 1M LiCl (50 μL, 0.05 mmol) was added to the solution in a 1-dram vial. The reaction solution was allowed to evaporate under a nitrogen atmosphere. After approximately 4-8 days green crystals were observed.

Note: A nominally $U(OH)_4$ precipitate was also dissolved in 0.5 M HCl to investigate the role of acid concentration for this phase. The phase isolated at 0.5 M HCl was characterized using PXRD (Figure S12) and is discussed in detail herein.

Synthesis of, $U(H_2O)_4Cl_4$ (2) was synthesized via the evaporation of 4 M HCl. The uranium stock solution was synthesized by dissolving UCl_4 (0.108 g, 0.28 mmol) in water (1 mL). An aliquot of concentrated NH_4OH (300 μL) was then added, causing a green solid to precipitate. The mixture was then centrifuged for 5 minutes at 4500 rpm in a 50 mL falcon tube and the supernatant was discarded. The resulting precipitate was washed with 1 mL of water and this process was repeated until the precipitate had been washed three times. The green precipitate was then dissolved in 1 mL of 4 M HCl. Aliquots from this acidic $U_{(aq)}$ stock solution (100 μL, 0.0262 mmol U, 6.26 mg U) were pulled and combined with 400 μL 4M HCl. 1M NaCl (30 μL, 0.03 mmol) as well as 1 M NaOH (20 μL, 0.02 mmol) was added to the solution in a 1-dram vial. The reaction solution was allowed to evaporate under a nitrogen atmosphere. After approximately 4-8 days green crystals were observed.

Synthesis of, **$U(H_2O)_4Cl_4\cdot KCl$, (3)** was synthesized via the evaporation of 4 M HCl. The uranium stock solution was synthesized by dissolving UCl_4 (0.108 g, 0.28 mmol) in water (1 mL).An aliquot of concentrated NH_4OH (300μL) was then added, causing a green solid to precipitate. The mixture was then centrifuged for 5 minutes at 4500 rpm in a 50 mL falcon tube and the supernatant was discarded. The resulting precipitate was washed with 1 mL of water and this process was repeated until the precipitate had been washed three times. The green precipitate was then

dissolved in 1 mL of 4 M HCl. Aliquots from this acidic $U_{(aq)}$ stock solution (100 µL, 0.0262 mmol U, 6.26 mg U) were pulled and combined with 400 µL 4 M HCl. 1 M KCl (30 µL, 0.03 mmol) as well as 1 M KOH (20 µL, 0.02 mmol) was added to the solution in a 1-dram vial. The reaction solution was allowed to evaporate under a nitrogen atmosphere. After approximately 4-8 days green crystals were observed.

Synthesis of, Rb_2UCl_6 (4) was synthesized via evaporation. A uranium stock solution was prepared by dissolving UCl_4 (0.108 g, 0.284 mmol) in water (1 mL). An aliquot of concentrated NH_4OH (300 µL) was then added, causing a green solid to precipitate. The mixture was then centrifuged for 5 minutes at 4500 rpm in a 50 mL falcon tube and the supernatant was discarded. The resulting precipitate was washed with 1 mL of water and this process was repeated until the precipitate had been washed three times. The green precipitate was then dissolved in 1 mL of 4 M HCl. Aliquots from this acidic $U_{(aq)}$ stock solution (100 µL, 0.0262 mmol U, 6.26 mg U) were pulled and combined with 400 µL 4 M HCl. 1 M RbCl (50 µL, 0.05 mmol) was added to the solution in a 1-dram vial. The reaction solution was allowed to evaporate under a nitrogen atmosphere. After approximately 4-8 days green crystals were observed.

Synthesis of, Cs_2UCl_6 (5) was synthesized via evaporation. A uranium stock solution was prepared by dissolving UCl_4 (0.108 g, 0.284 mmol) in water (1 mL). An aliquot of concentrated NH_4OH (300 µL) was then added, causing a green solid to precipitate. The mixture was then centrifuged for 5 minutes at 4500 rpm in a 50 mL falcon tube and the supernatant was discarded. The resulting precipitate was washed with 1 mL of water and this process was repeated until the precipitate had been washed three times. The green precipitate was then dissolved in 1 mL of 4 M HCl. Aliquots from this acidic U(aq) stock solution (100 µL, 0.0262 mmol U, 6.26 mg U) were pulled and combined with 400 µL 4 M HCl. 1M CsCl (50 µL, 0.05 mmol) was added to the solution in a 1-dram vial. The reaction solution was allowed to evaporate under a nitrogen atmosphere. After approximately 4-8 days green crystals were observed.

2. CRYSTALLOGRAPHIC REFINEMENT DETAILS OF COMPOUNDS **1**–**4**.

Note: Crystallographic refinement details can be found in the "experimental refinement section" of the crystallographic information files (CIFs). Details are also provided below.

Compound 1

A structural model consisting of an anionic U4 cluster, 5 Li cations, and 10 lattice water molecules was developed. Based on the crystallographic model, there are two possible formulas for compound **1**: $Li_6[U_4(\mu_3\text{-}O)_2Cl_{18}(H_2O)_2]\cdot10H_2O$, and $Li_5[U_4((\mu_3\text{-}O)/OH)Cl_{18}(H_2O)_2]\cdot10H_2O$. We considered the possibility that the bridging site (O1) was a substitionally disordered oxo/hydroxo, which would then charge balance the model with only five Li cations. A potential disordered hydroxyl H atom could not be located in the difference map and bond valence summation value for O1(see Table S1) was consistent with O^{2-}. Based on these considerations, we have formulated the compound with six Li cations though it could not be located in the difference map. The H atoms for several of the water molecules were located in the difference map and the O-H distances were restrained to be 0.88 (esd 0.01 Å). H atoms for two of the lattice water molecules could not be located in the difference map and thus were left off of the model. H atom U's were assigned as 1.5 times carrier U_{eq}.

Compound 2

A structural model consisting of one half of the target complex per asymmetric unit was developed. Assignment of Cl/H_2O sites to fill out the U coordination sphere was based on the size of electron density peaks in the difference map, bond distances to the U metal center, and the resulting thermal parameters of the Cl/O atom assignment. Additionally, residual density peaks were located near the O sites in the expected positions for hydrogen atoms of coordinated water molecules. The water H atoms were located in the difference map. The O-H distances were restrained to be 0.88 (esd 0.01 Å). There are several large residual density peak in the difference map near the uranium metal center. This is likely attributed to unaccounted twinning. However, attempts to process the data as either a non-merohedral or pseudo-merohedral twin resulted in very poor structural models.

Compound 3

A structural model consisting of the target ionic complex was developed. Assignment of Cl/H_2O sites to fill out the U coordination sphere was based on the size of electron density peaks in the difference map, bond distances to the U metal center, and the resulting thermal parameters of the Cl/O atom assignment. Additionally, residual density peaks were located near the O sites in the expected positions for hydrogen atoms of coordinated water molecules. The water H atoms were located in the difference map. The O-H distances were restrained to be 0.88 (esd 0.01 Å). Water H atom U's were assigned as 1.5 times U_{eq} of the carrier atom.

Compound 4

A structural model consisting of one third of the target complex per asymmetric unit was developed. The datum crystal was processed as a two component non-merohedral twin, however, the structure solution and refinement used reflections from only the primary domain.

3. ORTEP DIAGRAMS OF COMPOUNDS **1–4**

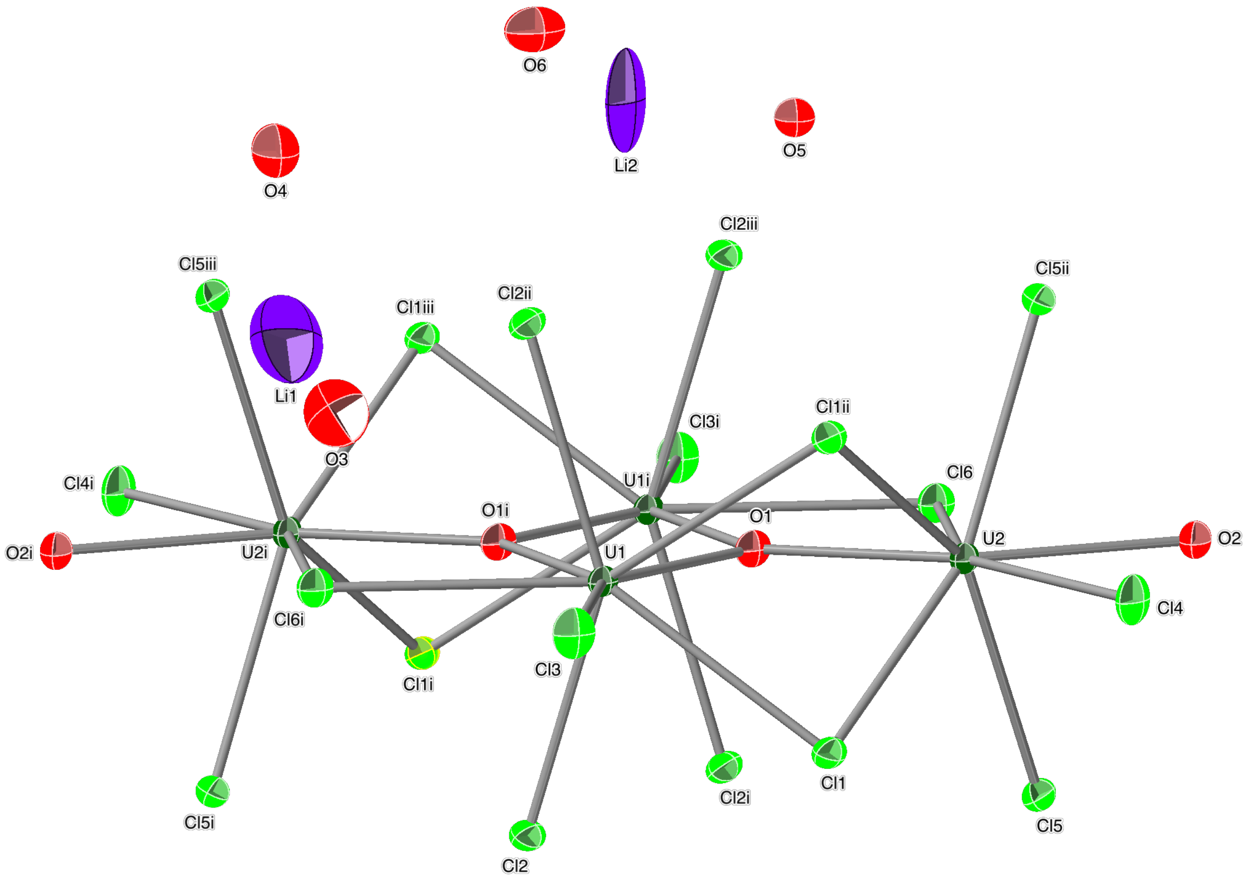

Figure S1. Thermal ellipsoid plot (50% probability level) of compound **1**, $Li_6[U_4(\mu_3\text{-}O)_2Cl_{18}(H_2O)_2]\cdot 10\ H_2O$, at 100 K. Color code: U, dark green; O, red; Cl, green; Li, purple. Hydrogen atoms are omitted for clarity. Symmetry equivalent atoms were generated through their respective symmetry elements (i = 1-x, y, 1-z), (ii = x, 1-y, z), (iii = 1-x, 1-y, 1-z).

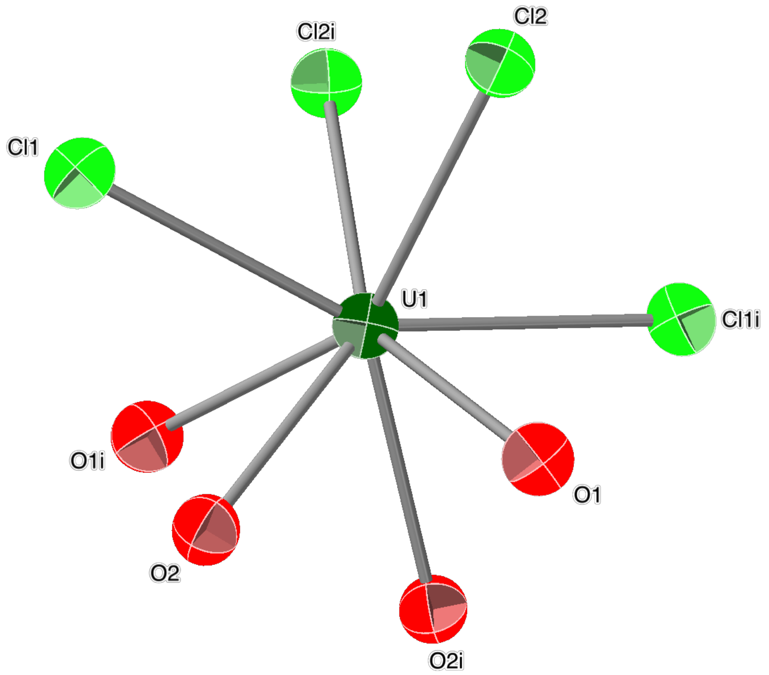

Figure S2. Thermal ellipsoid plot (50% probability level) of compound **2**, ($U(H_2O)_4Cl_4$), at 100 K. Color code: U, dark green; O, red; Cl, green. Hydrogen atoms are omitted for clarity. Symmetry equivalent atoms were generated through their respective symmetry elements (i = -x, y, ½-z)

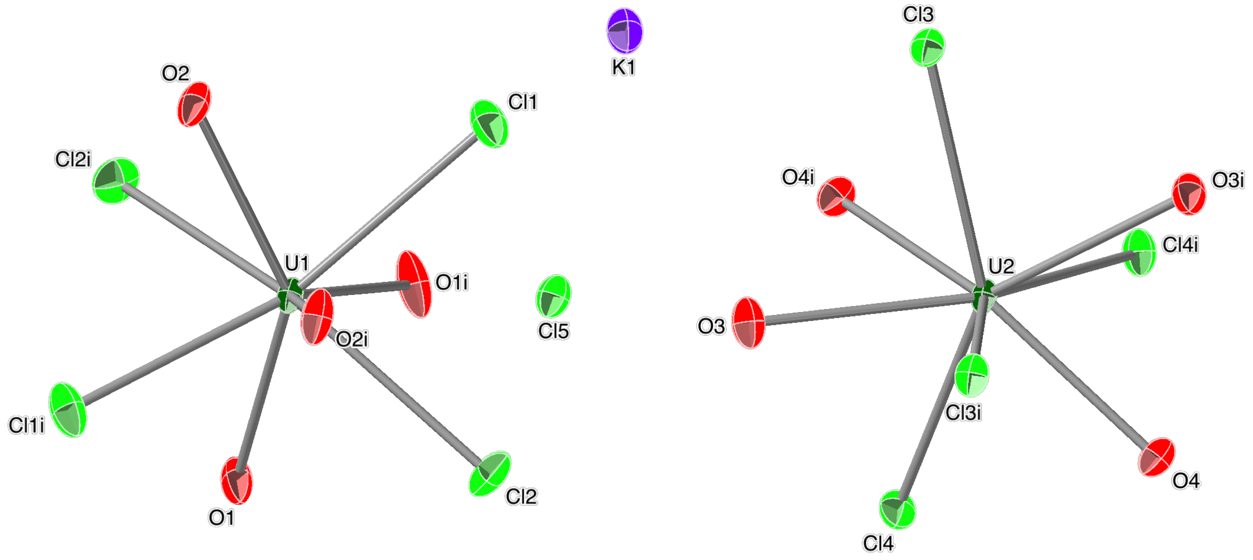

Figure S3. Thermal ellipsoid plot (50% probability level) of compound **3**, ($U(H_2O)_4Cl_4$)·KCl, at 100 K. Color code: U, dark green; O, red; Cl, green; K, purple. Hydrogen atoms are omitted for clarity. Symmetry equivalent atoms were generated through their respective symmetry elements (i = -x, y, ½-z.)

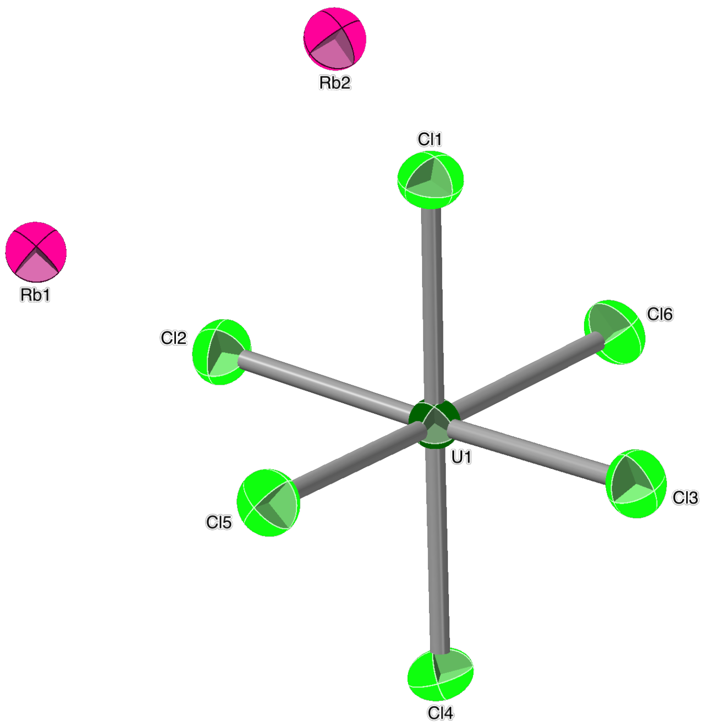

Figure S4. Thermal ellipsoid plot (50% probability level) of compound **4**, Rb_2UCl_6, at 100 K. Color code: U, dark green; Cl, green; Rb, pink.

4. BOND VALENCE SUMMATION VALUES FOR **1**.

Bond valence summation values were calculated using the equation $\nu_{ij} = \exp[(R_{ij}-d_{ij})/b)$ where ν_{ij} is the overall individual valence for a particular atom, d_{ij} is the bond distance between atoms *i* and *j,* R_{ij} is the associated bond valence parameter, and b is a constant. All of the individual valences were summed to give the overall valence for particular atoms.

U^{4+} parameters for Bond Valence = 2.112 for O.

b = 0.37

Table S1. Bond Valence Summation values for **1**, using R_{ij} (U^{IV}). Parameters were obtained from: Brese N. E; O'Keeffe M., Bond-Valence Parameters for Solids. Acta Cryst. 1991, B47, 192-197.

U1		
ATOM	DISTANCE	BOND VALENCE
O1	2.297	0.57773
O1	2.297	0.57773
O1	2.297	0.57773
O1	2.297	0.57773
O2	2.368	0.4768561
O2	2.368	0.4768561
O2	2.368	0.4768561
O2	2.368	0.4768561
	Sum	4.2183443

O1		
ATOM	DISTANCE	BOND VALENCE
U1	2.244	0.69994273
U2	2.177	0.83889001
U3	2.274	0.64543044
	Sum	2.18426318

5. PACKING DIAGRAMS for **1-4**.

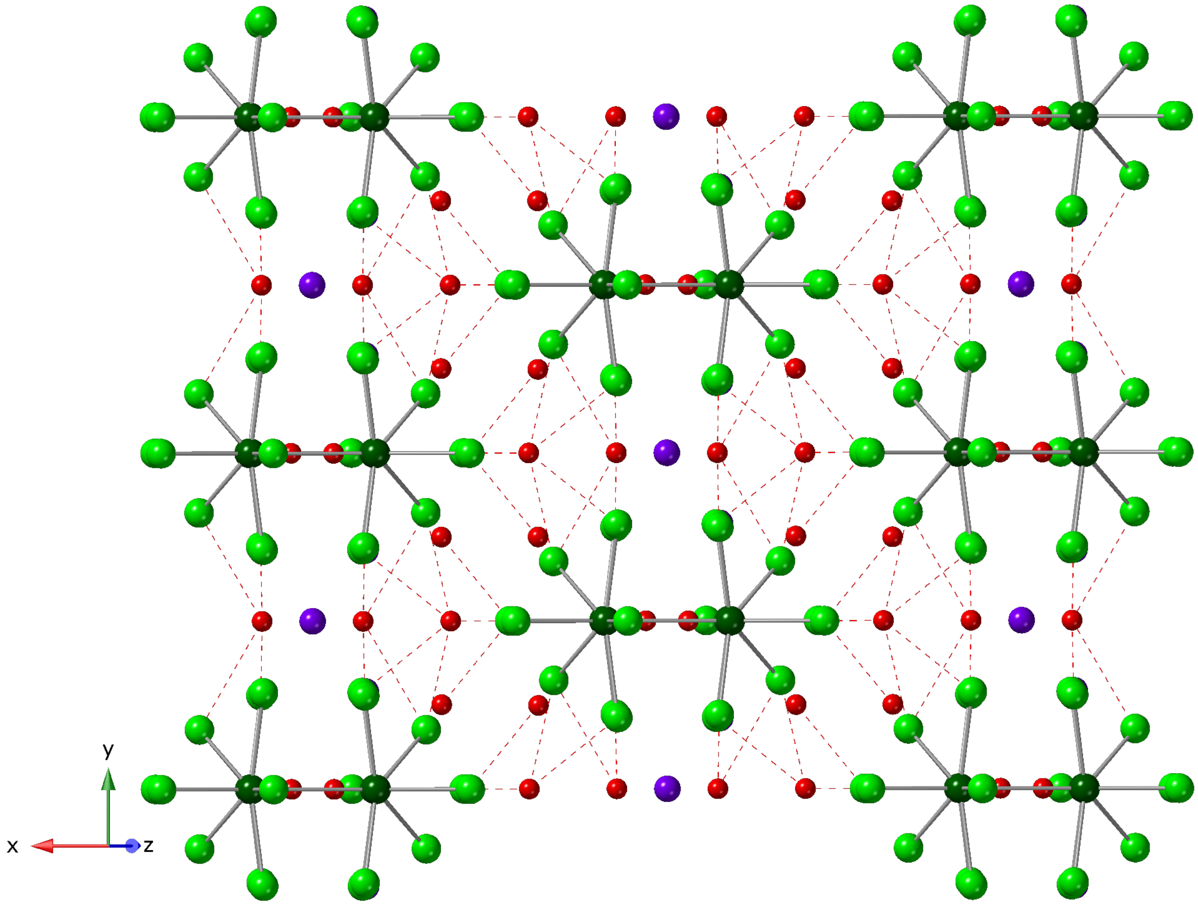

Figure S5. Packing diagram of compound **1,** $Li_6[U_4(\mu_3\text{-}O)_2Cl_{18}(H_2O)_2]\cdot 10H_2O$, showing the hydrogen bonding along the [100], and [010] axes, propagating this structural unit into an overall 3-dimensional supramolecular unit. Color code: U, dark green; O, red; Cl, green; Li, purple. Hydrogen atoms are omitted for clarity.

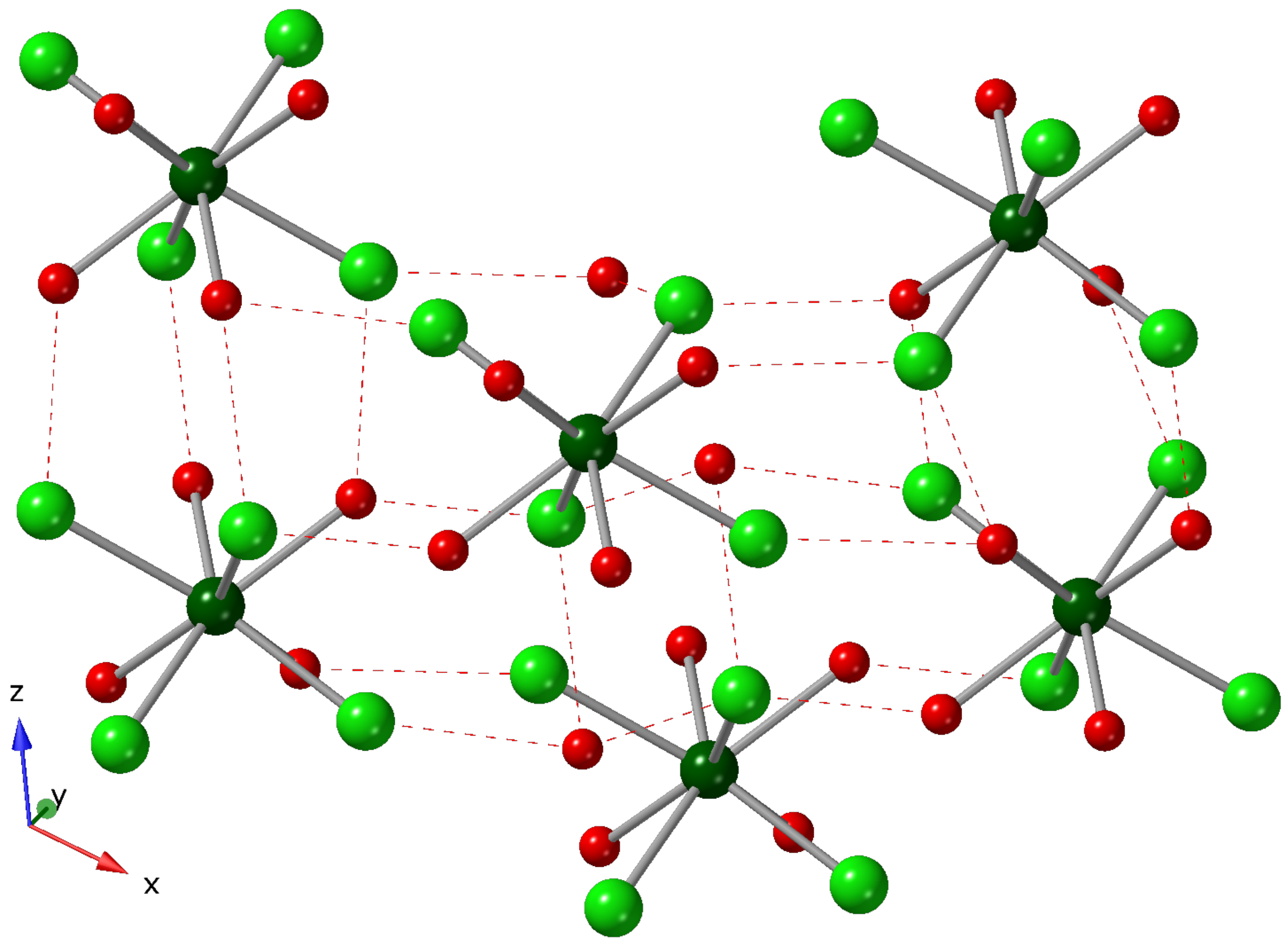

Figure S6. Packing diagram for compound **2,** ($U(H_2O)_4Cl_4$), showing the hydrogen bonding along the [001], and [100] axes, propagating this structural unit into an overall 3-dimensional supramolecular unit. Color code: U, dark green; O, red; Cl, green. Hydrogen atoms are omitted for clarity.

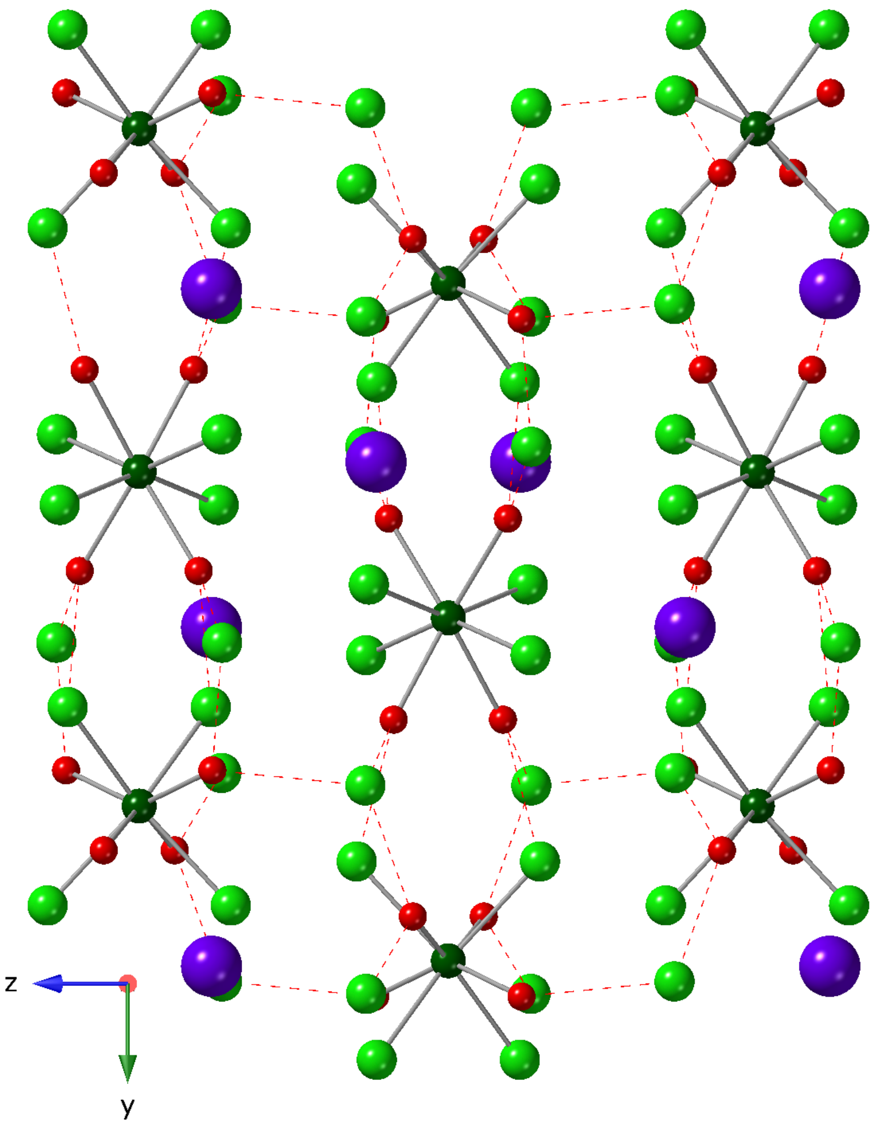

Figure S7. Packing diagram for compound **3,** $(U(H_2O)_4Cl_4)\cdot KCl$, showing the hydrogen bonding along the [010], and [001] axes, propagating this structural unit into an overall 3-dimensional supramolecular unit. Color code: U, dark green; O, red; Cl, green; K, purple. Hydrogen atoms are omitted for clarity.

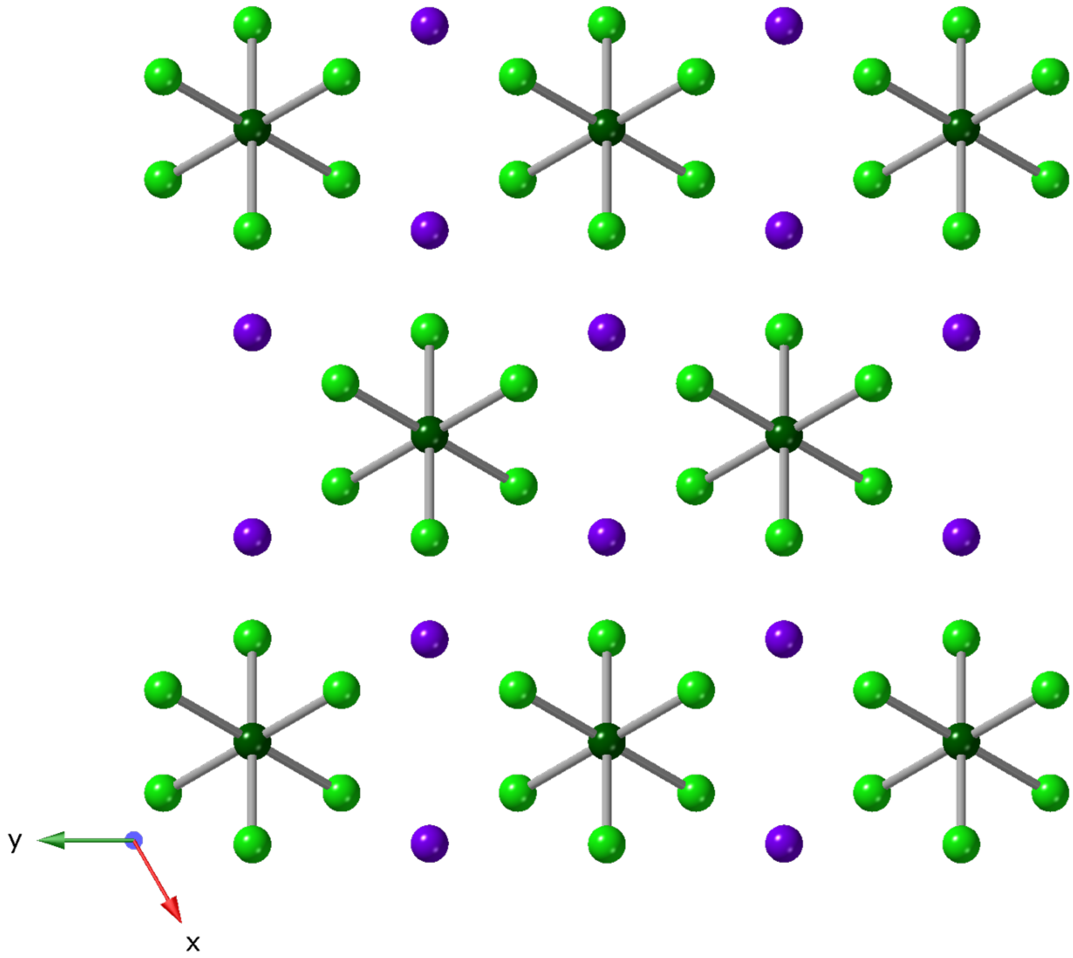

Figure S8. Packing diagram for compound **4,** Rb_2UCl_6**.** Color code: U, dark green; O, red; Cl, green; Rb, purple.

6. POWDER X-RAY DIFFRACTION PATTERNS for **1**–**5**.

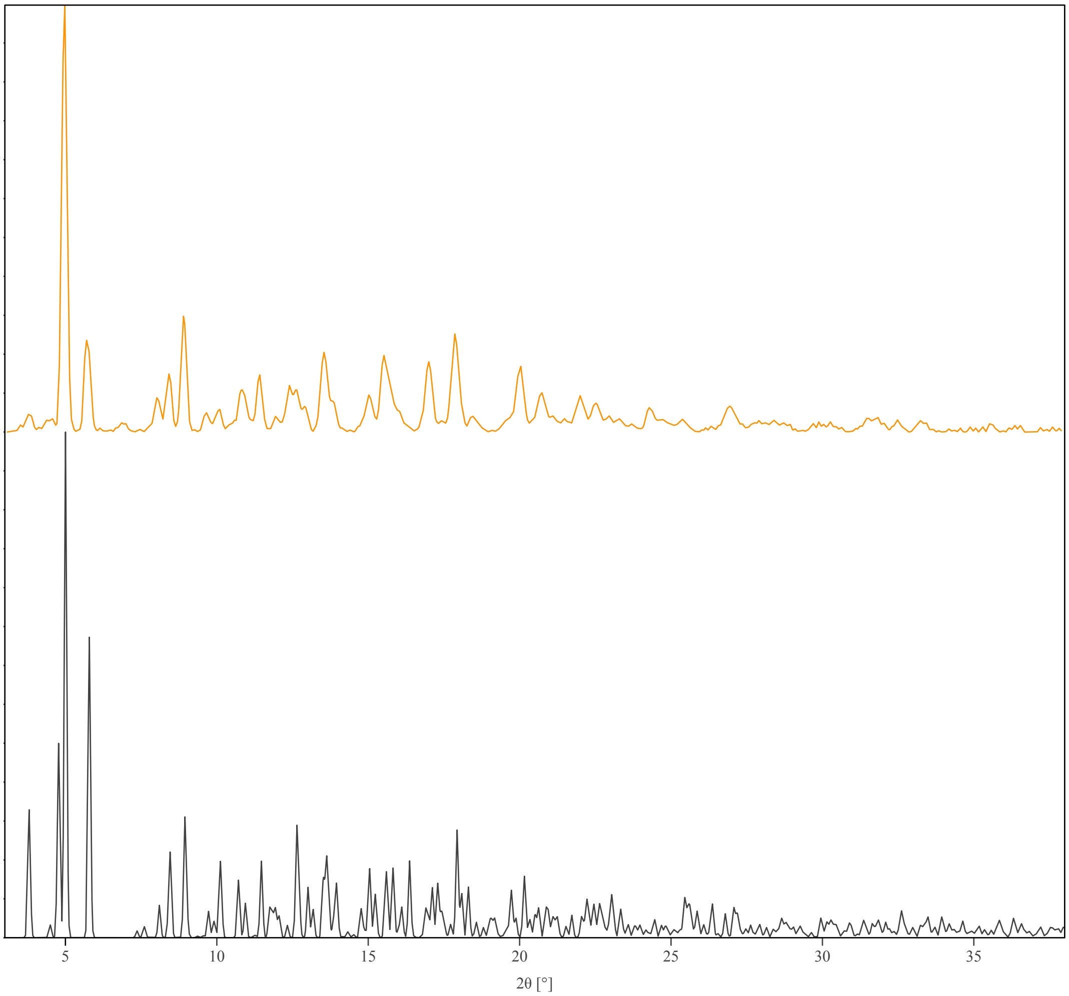

Figure S9. Powder X-ray diffraction pattern (collected with Cu Kα radiation) observed for the reaction product from which **1** was isolated (orange) overlaid with the pattern calculated from the single crystal structure of **1** at 100 K (black).

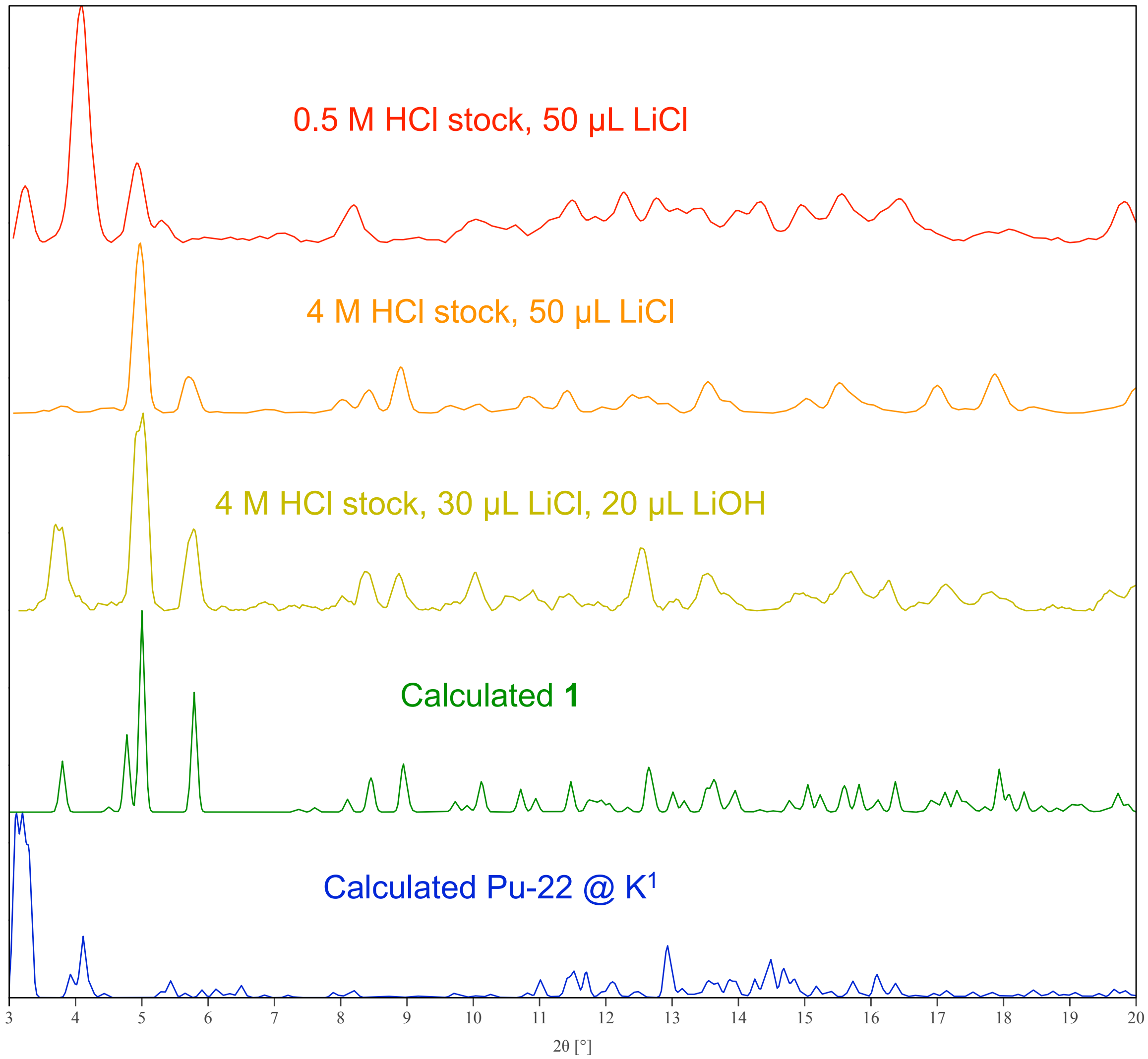

Figure S10. Powder X-ray diffraction patterns (collected with Cu Kα radiation) observed for the reaction product from which **1** was isolated (orange) overlaid with the pattern calculated from the single crystal structure of **1** at 100 K (green). In an attempt to assign other peaks, the experimental powder was compared to other calculated patterns. Red shows the experimental pattern collected from crystals of a reaction using a 0.5M HCl stock solution as well as 50 µL LiCl. Additionally yellow shows the experimental pattern collected from crystals of a reaction using a 4M HCl stock solution as well as 30 µL LiCl and 20 µL LiOH. Blue shows the calculated pattern from the single crystal structures of previously reported potassium $\{Pu_{22}\}$ cluster.[1]

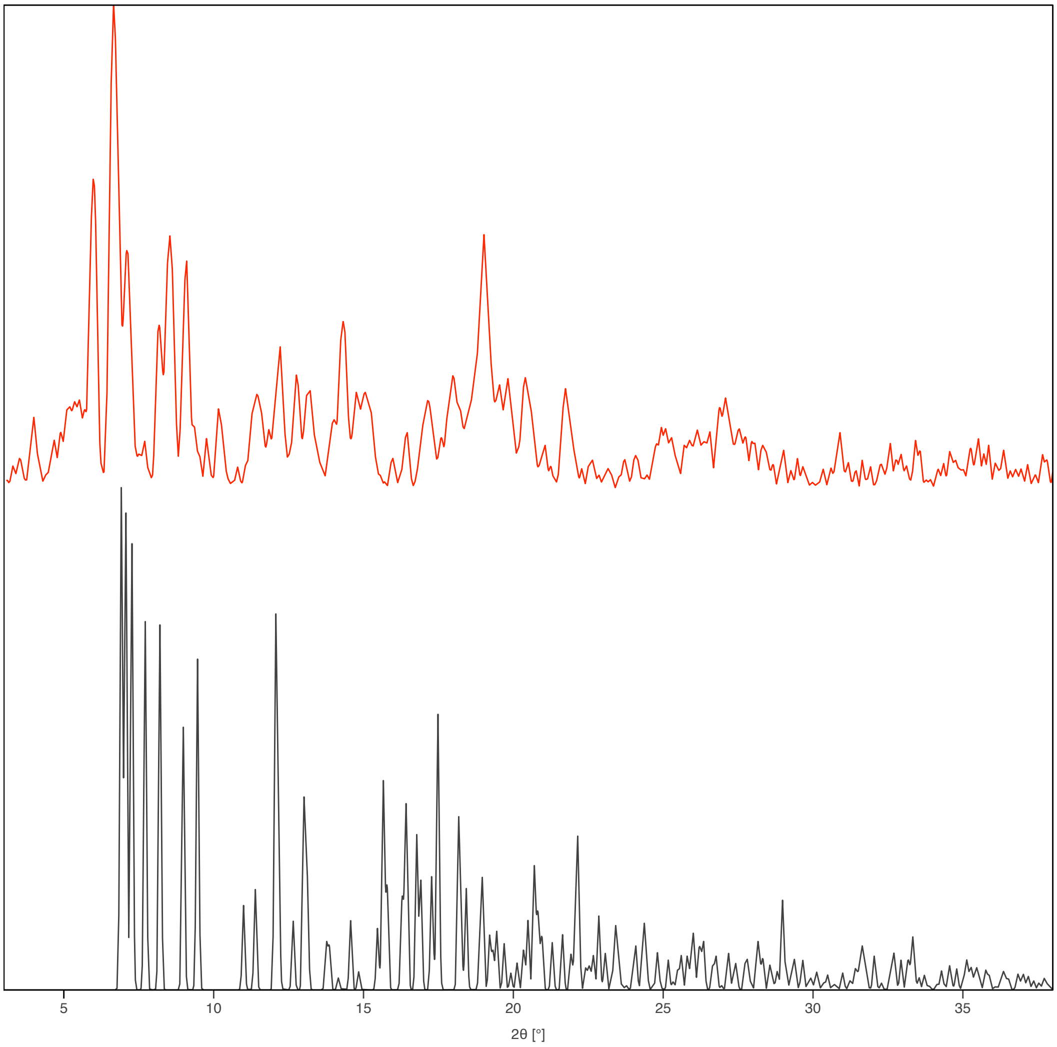

Figure S11. Powder X-ray diffraction pattern (collected with Cu Kα radiation) observed for the reaction product from which **2** was isolated (red) overlaid with the pattern calculated from the single crystal structure of **2** at 100 K (black).

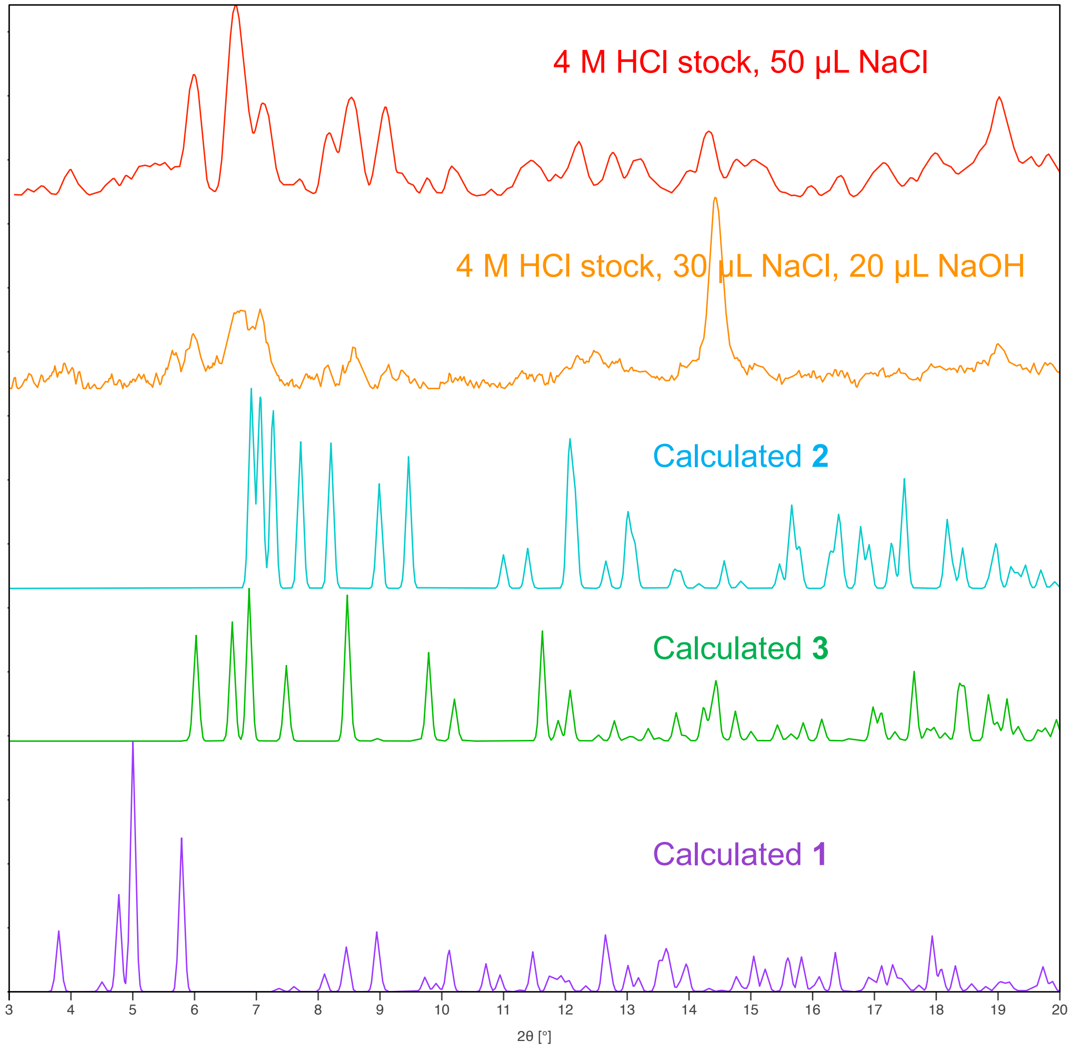

Figure S12. Powder X-ray diffraction pattern (collected with Cu Kα radiation) observed for the reaction product from which **2** was isolated (orange) overlaid with the pattern calculated from the single crystal structure of **2** at 100 K (teal). In an attempt to assign other peaks, the experimental powder was compared to other calculated patterns. Red shows the experimental pattern collected from crystals of a reaction using a 4M HCl stock solution as well as 50 µL NaCl. Green shows the pattern calculated from the single crystal structure of **3,** while purple shows the pattern calculated from the single crystal structure of **1.**

For **2** the powder x-ray diffraction pattern indicates that multiple phases are present. The major phases is identified from both the calculated CIF of **2**, and **3,** as both of these compounds share the same structural unit ($U(H_2O)_4Cl_4$) but with different coordination geometries and outer coordination sphere effects, it is likely that these phases could coprecipitate. At lower degrees two theta evidence suggests that the same tetrameric unit observed in compound **1** appears as a minor phase in compound **2**, while the major phases correspond to compounds **2** and **3**.

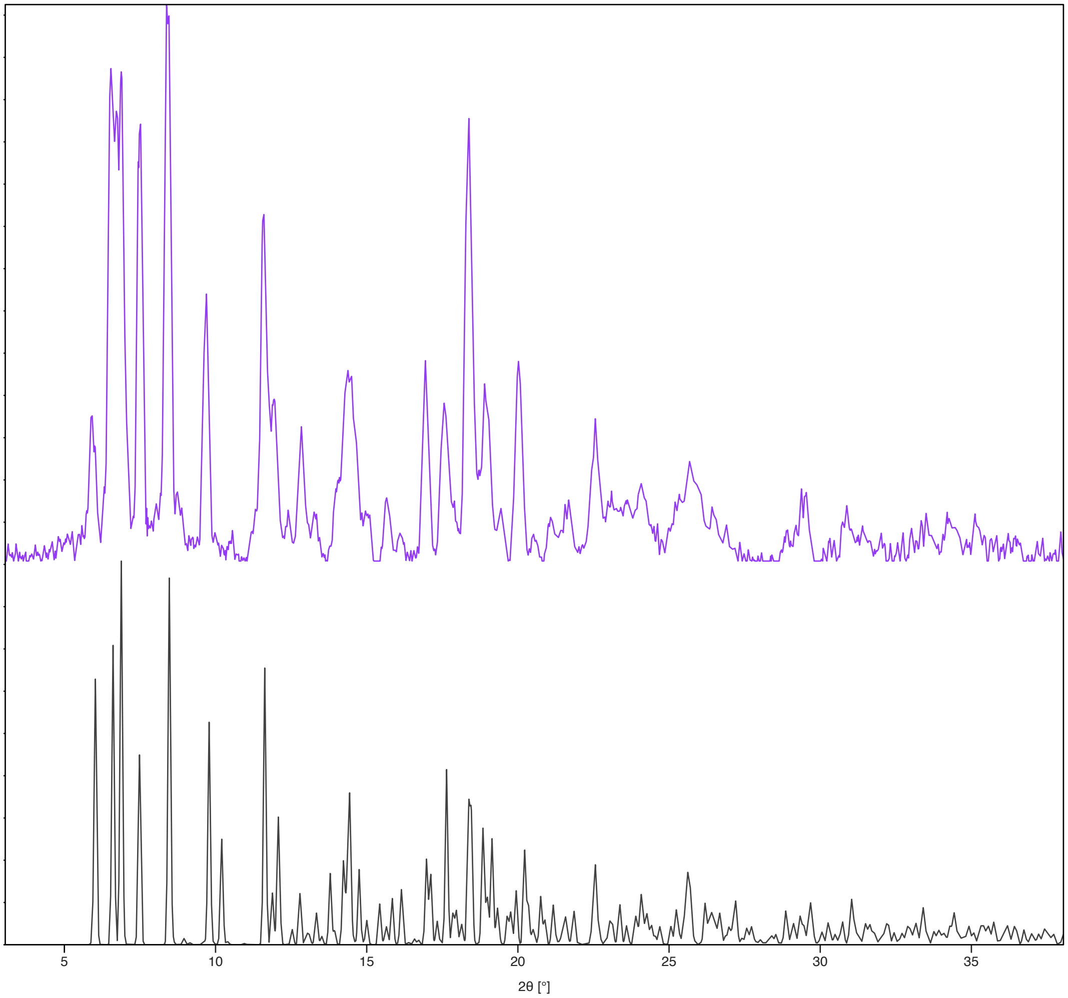

Figure S13. Powder X-ray diffraction pattern (collected with Cu Kα radiation) observed for the reaction product from which **3** was isolated (purple) overlaid with the pattern calculated from the single crystal structure of **3** at 100 K (black).

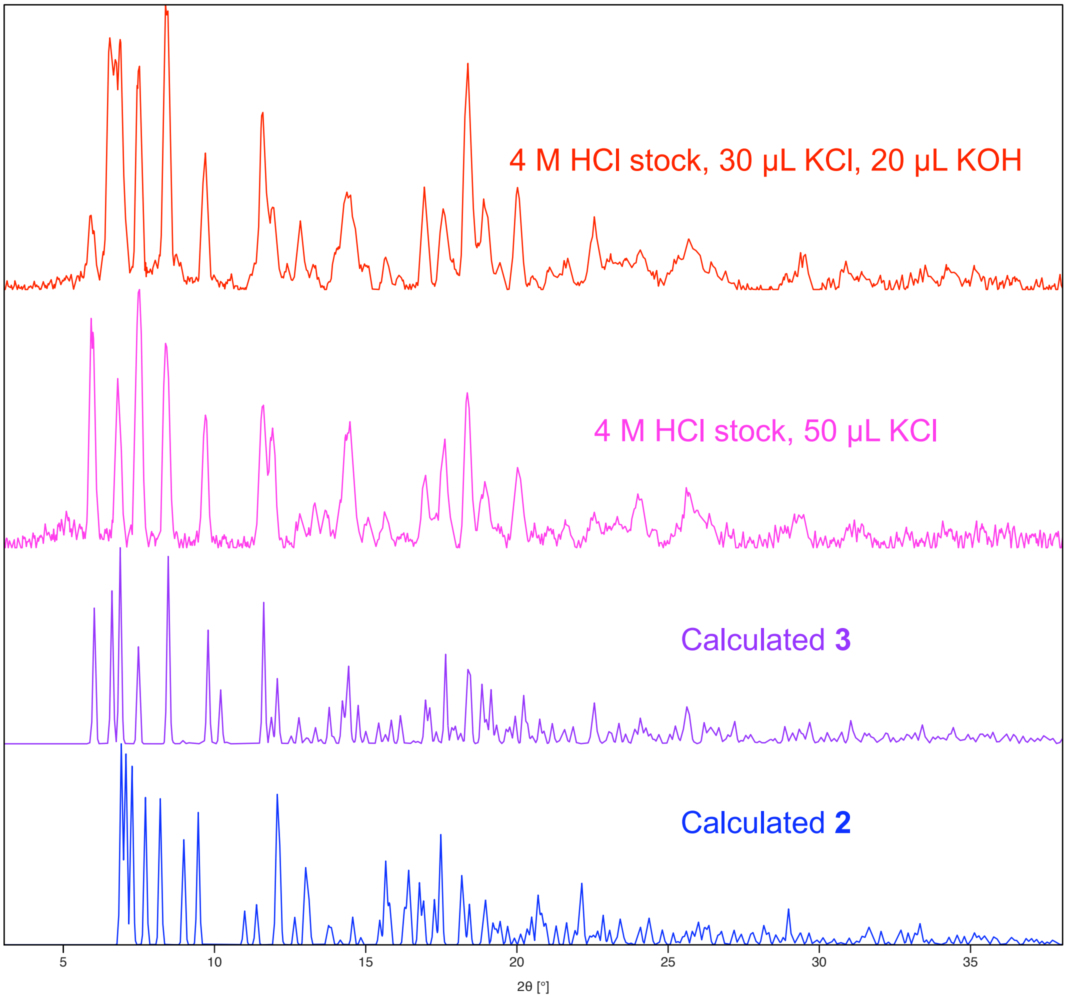

Figure S14. Powder X-ray diffraction pattern (collected with Cu Kα radiation) observed for the reaction product from which **3** was isolated (red) from crystals of a reaction using a 4M HCl stock solution as well as 30 µL KCl, and 20 µL KOH overlaid with the pattern calculated from the single crystal structure of **3** at 100 K (purple). Pink shows the experimental pattern collected from crystals of a reaction using a 4M HCl stock solution as well as 50 µL KCl. Additionally blue shows the pattern calculated from the single crystal structure of **2.**

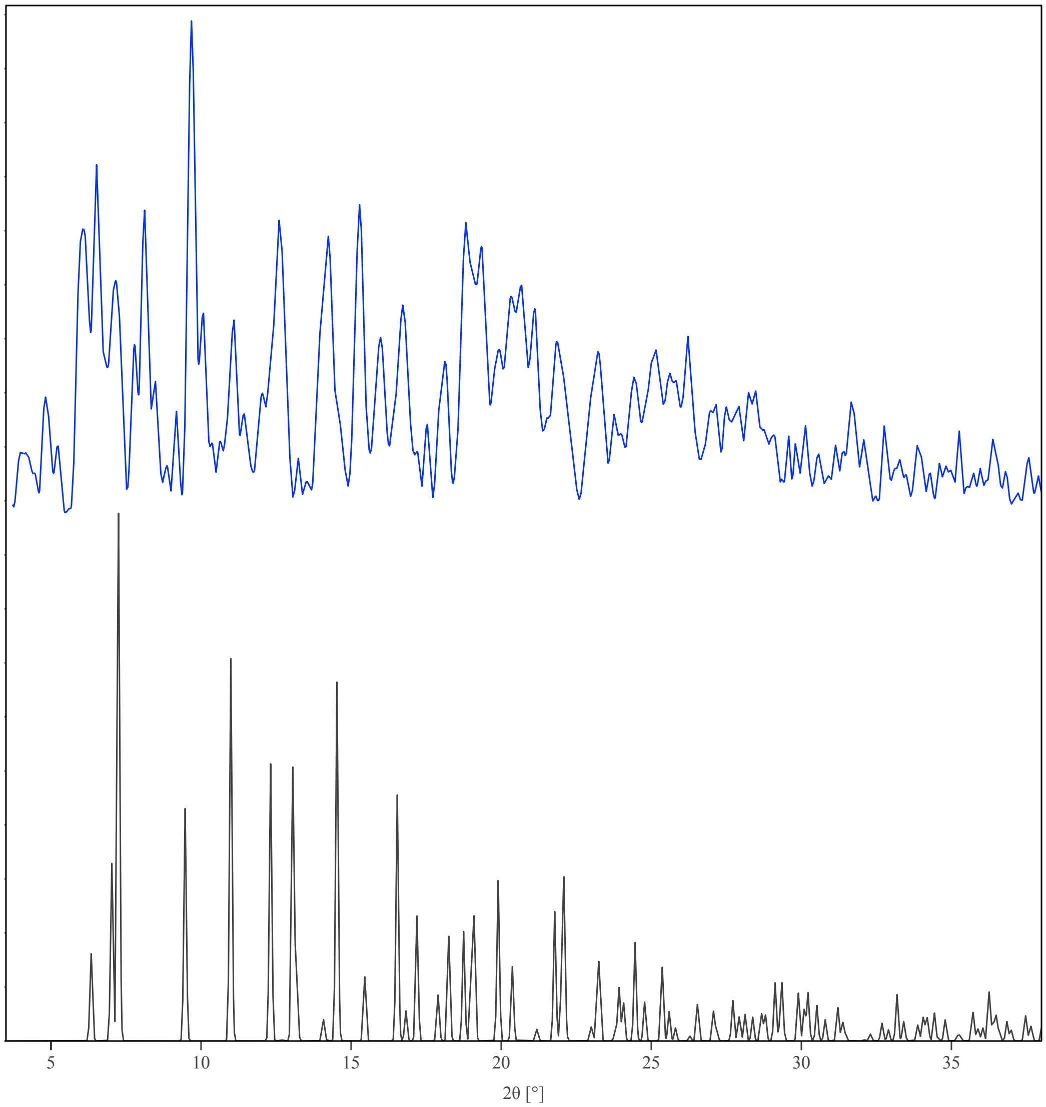

Figure S15. Powder X-ray diffraction pattern (collected with Cu Kα radiation) observed for the reaction product from which **4** was isolated (blue) overlaid with the pattern calculated from the single crystal structure of **4** at 100 K (black).

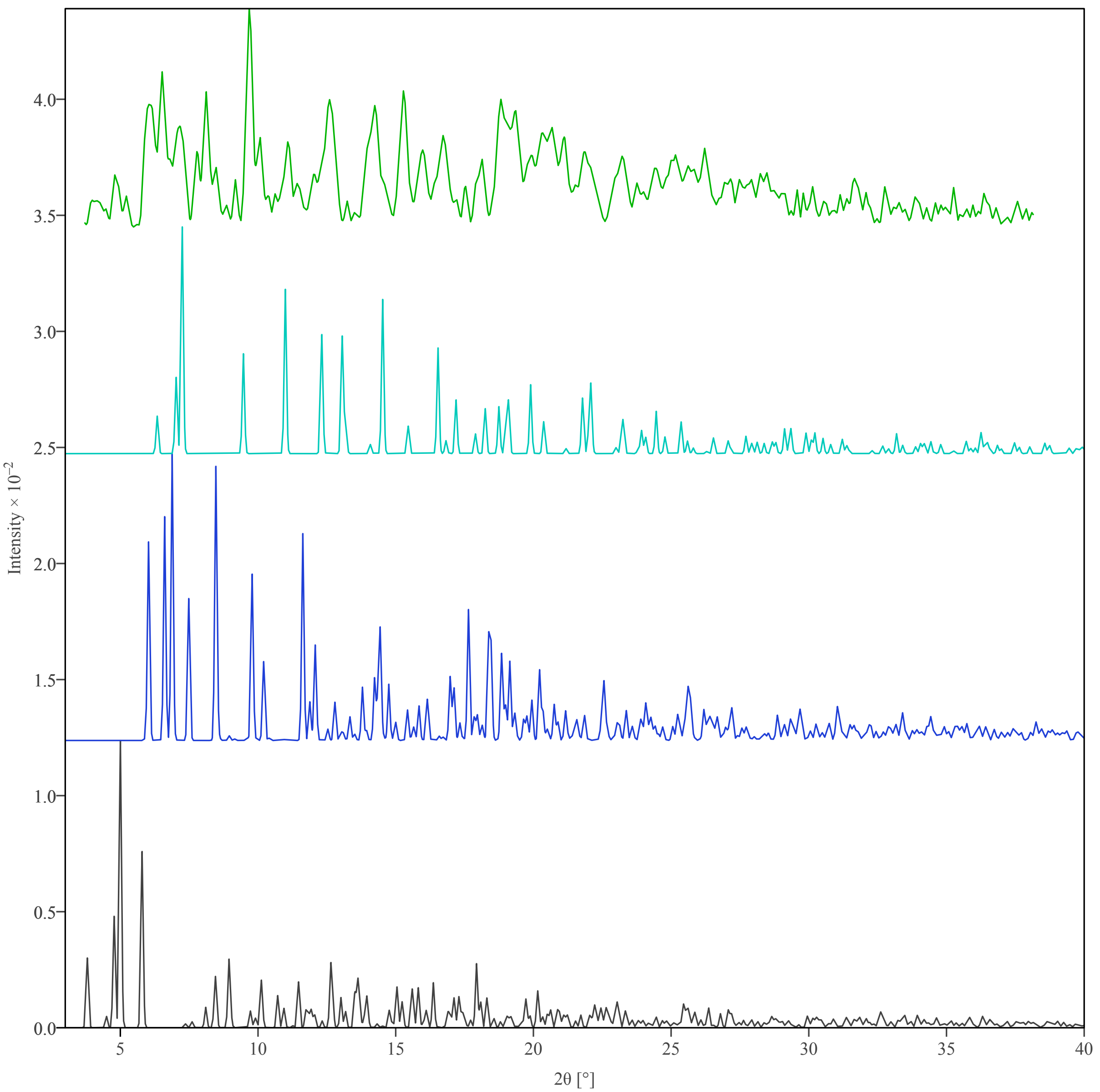

Figure S16. Powder X-ray diffraction pattern (collected with Cu Kα radiation) observed for the reaction product from which **4** was isolated (green) overlaid with the pattern calculated from the single crystal structure of **4** at 100 K (teal). In an attempt to assign other peaks, the experimental powder was compared to two other calculated patterns. Blue shows the pattern calculated from the single crystal structure of **3**, and black shows the pattern calculated from the single crystal structure of **1**. The supplemental power patterns help to ascertain the identity of minor phases

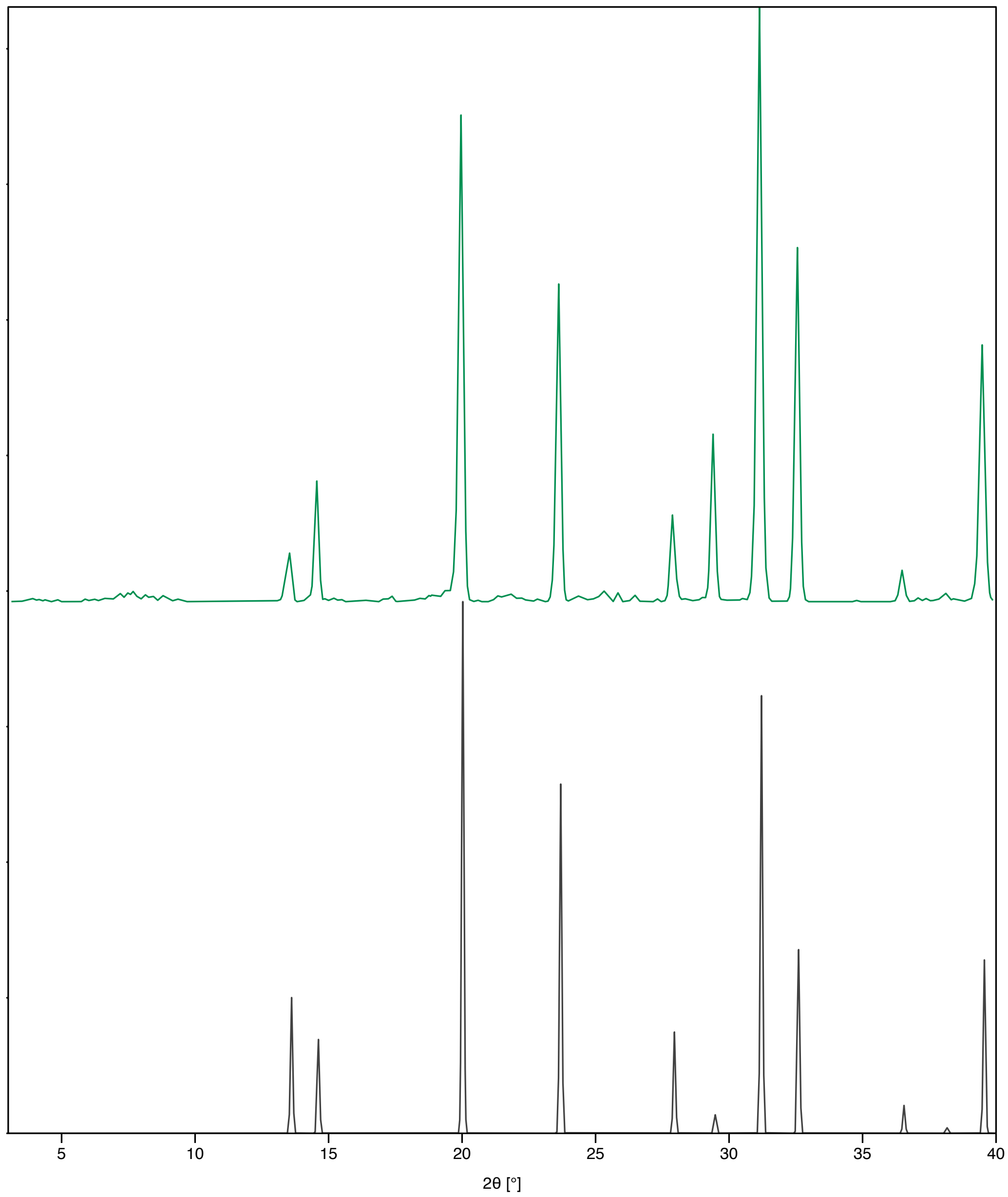

Figure S17. Powder X-ray diffraction pattern (collected with Cu Kα radiation) observed for the reaction product from which **5** was isolated (green) overlaid with the pattern calculated from the single crystal structure of **5** at 100 K (black).

7. RAMAN SPECTRA OF COMPOUNDS **1–5**.

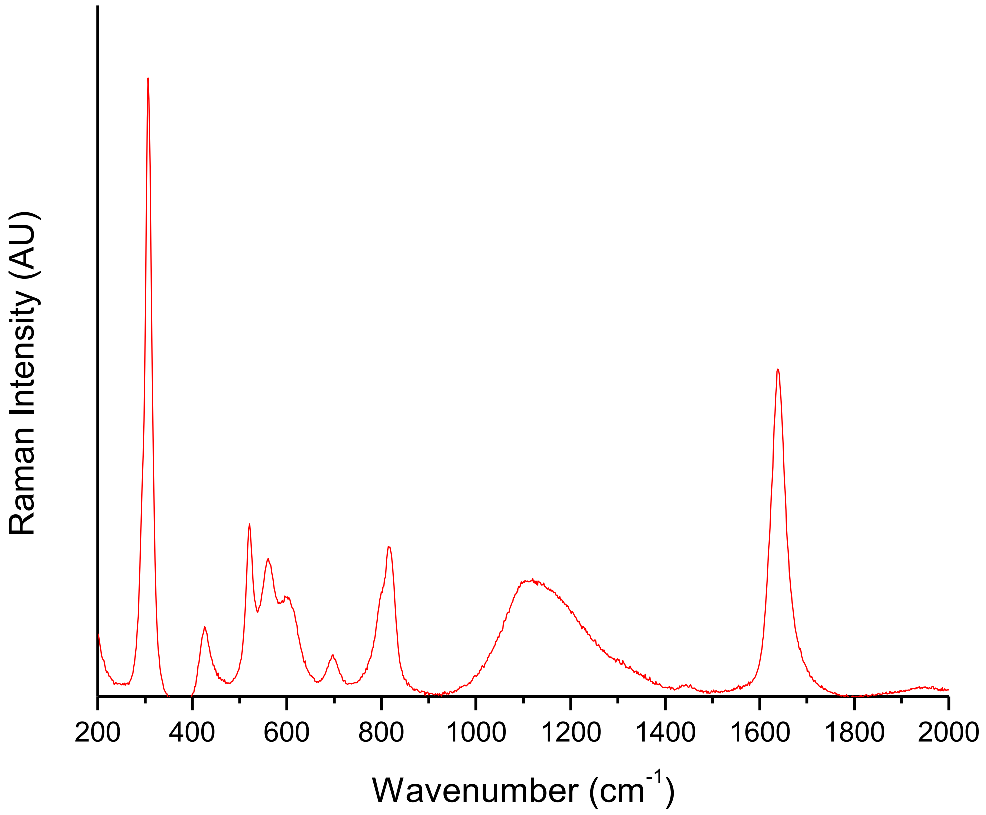

Figure S18. Raman spectrum of **1** (Raman =red). Raman: 306, 425, 520, 560, 595, 697, 814, 1120, 1638 cm^{-1}.

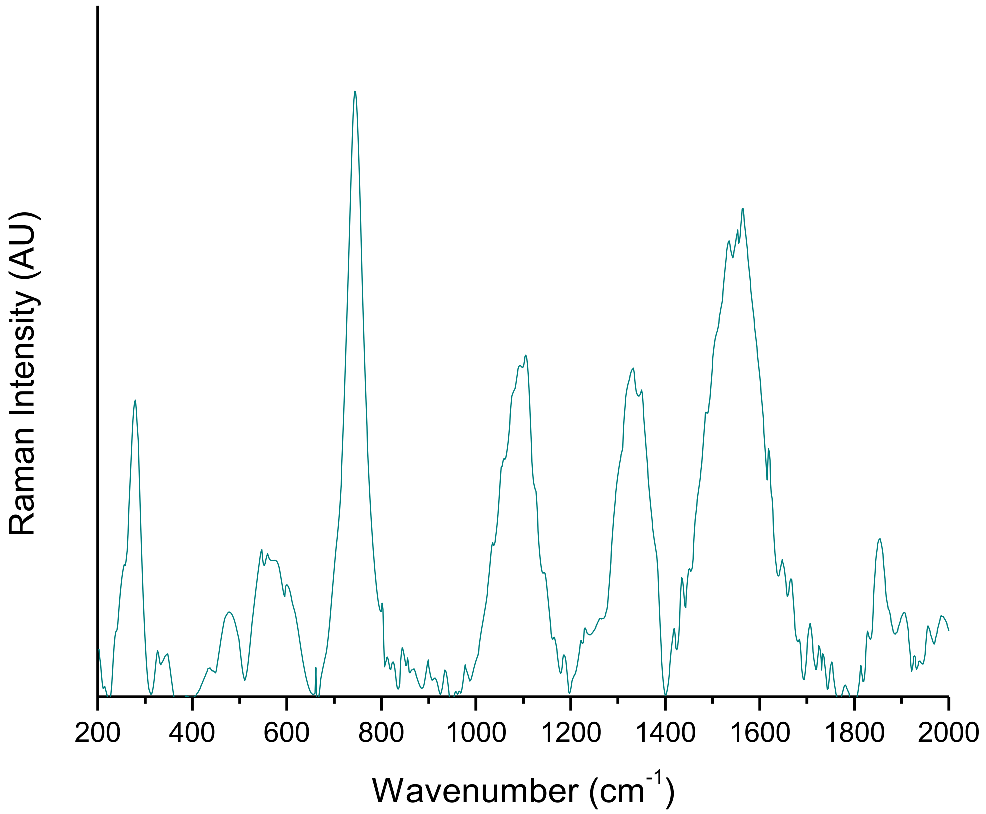

Figure S19. Raman (teal) spectra of **2**. Raman: 279, 347, 482, 559, 602, 745, 1107, 1334, 1566, 1857 cm^{-1}.

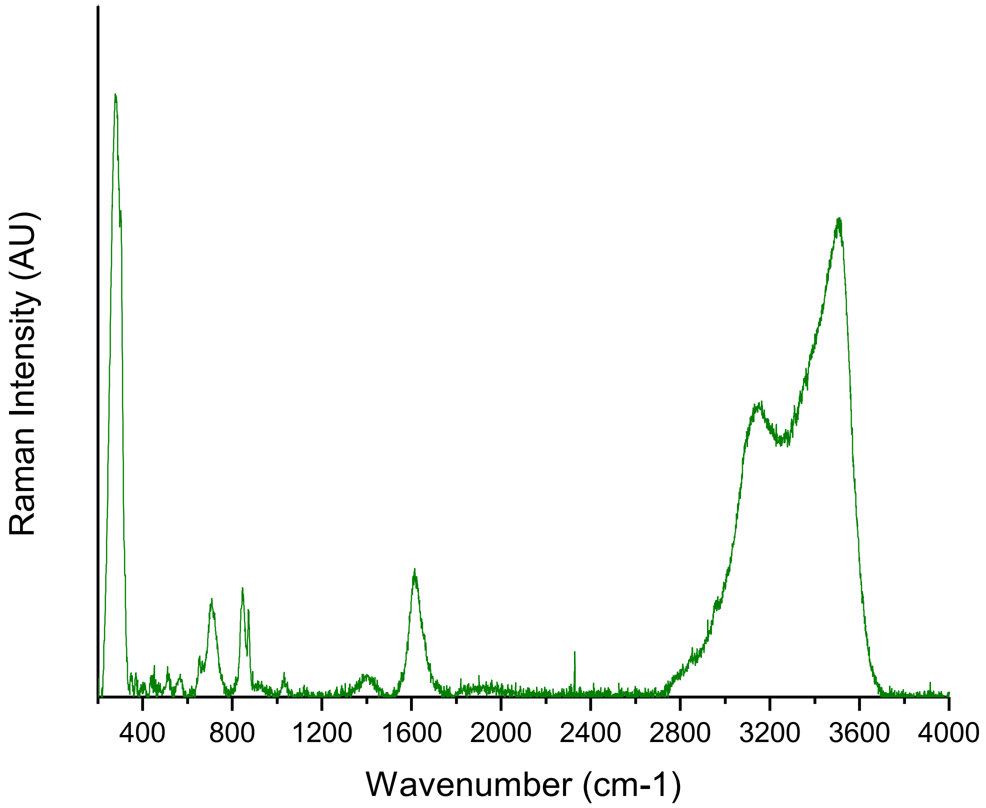

Figure S20. Raman (green) spectra of **3**. Raman: 276, 654, 708, 846, 871, 1031, 1399, 1614, 2329, 3152, 3511 cm^{-1}.

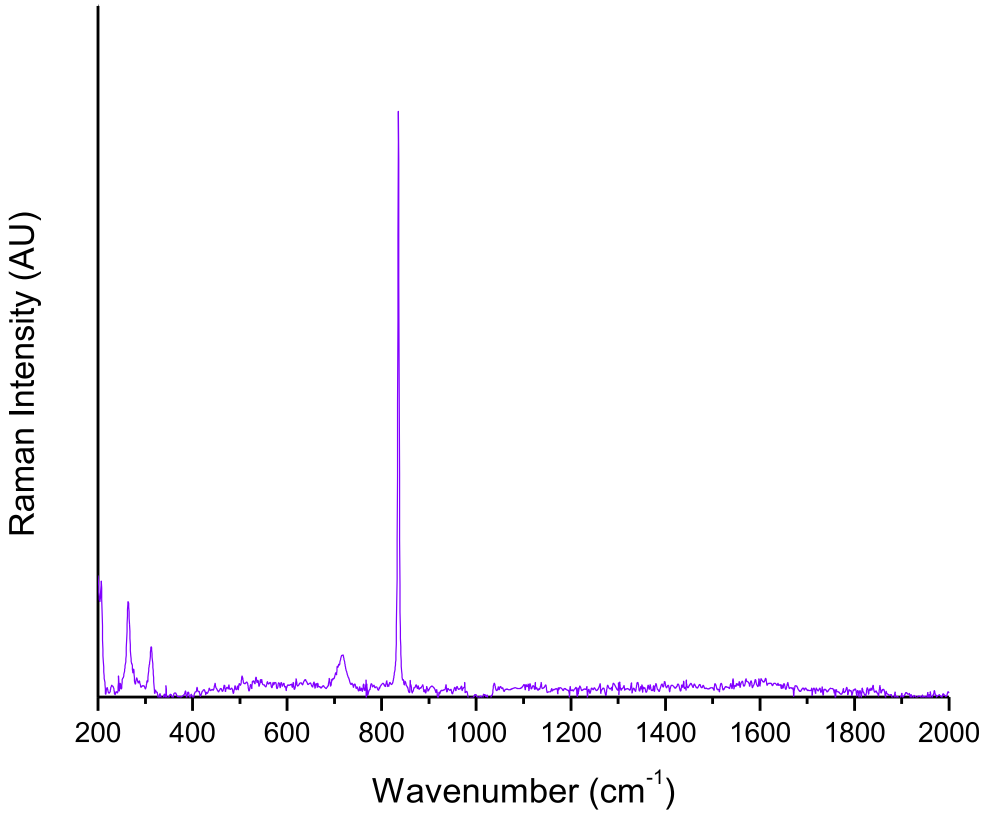

Figure S21. Raman (purple) spectra of **4**. Raman: 264, 312, 716, 835 cm^{-1}.

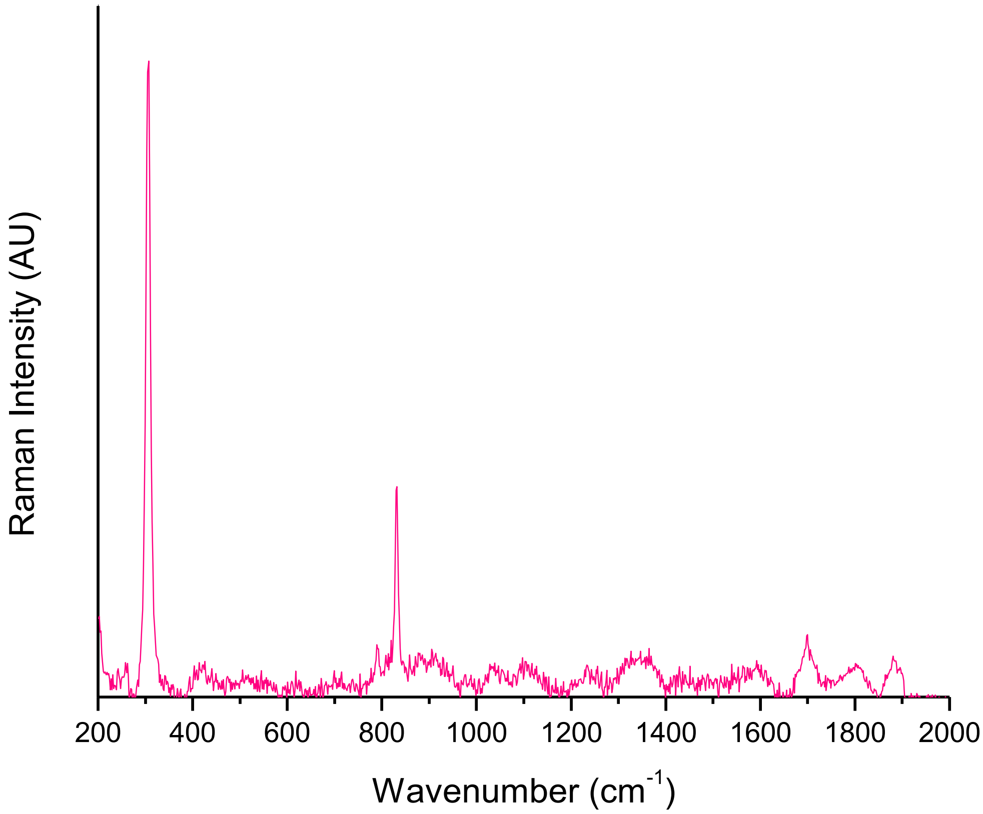

Figure S22. Raman (pink) spectra of **5**. Raman: 306, 831 cm^{-1}.

8. UV-Vis Absorption spectra of **1**–**5**

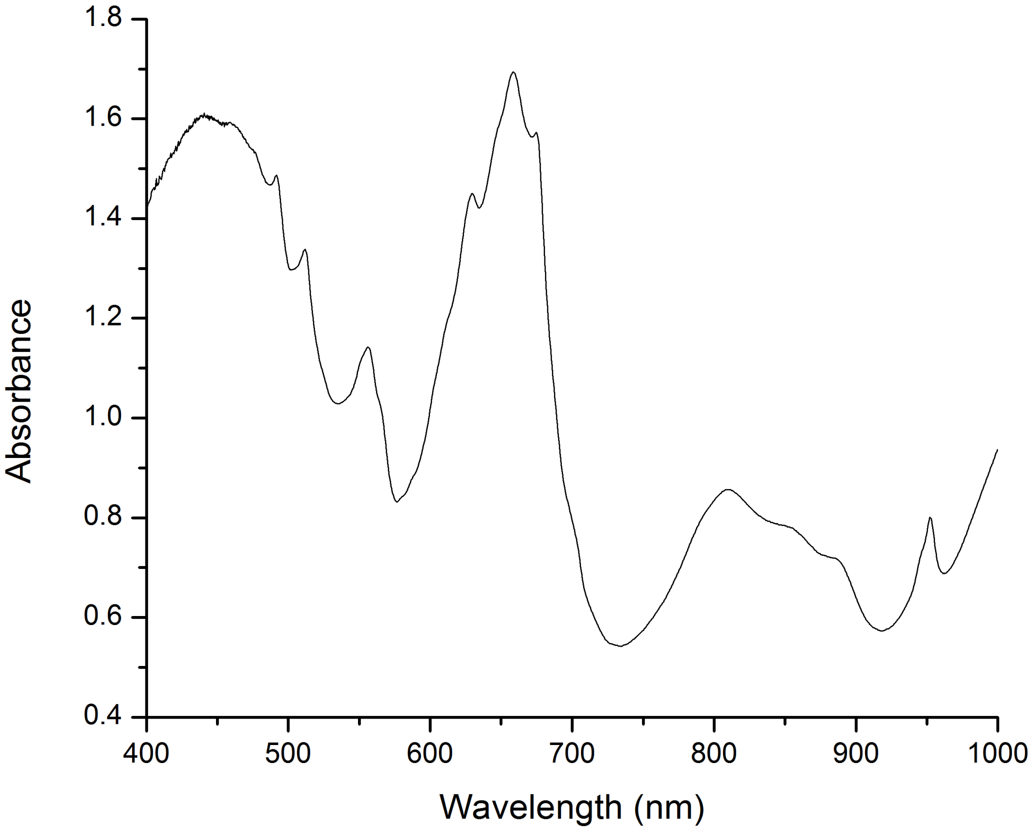

Figure S23. UV-Vis spectrum of **1**. Bands are observed at 495, 512, 555, 565, 586, 610, 629, 658, 674, 700, 810, 850, 885, 950.

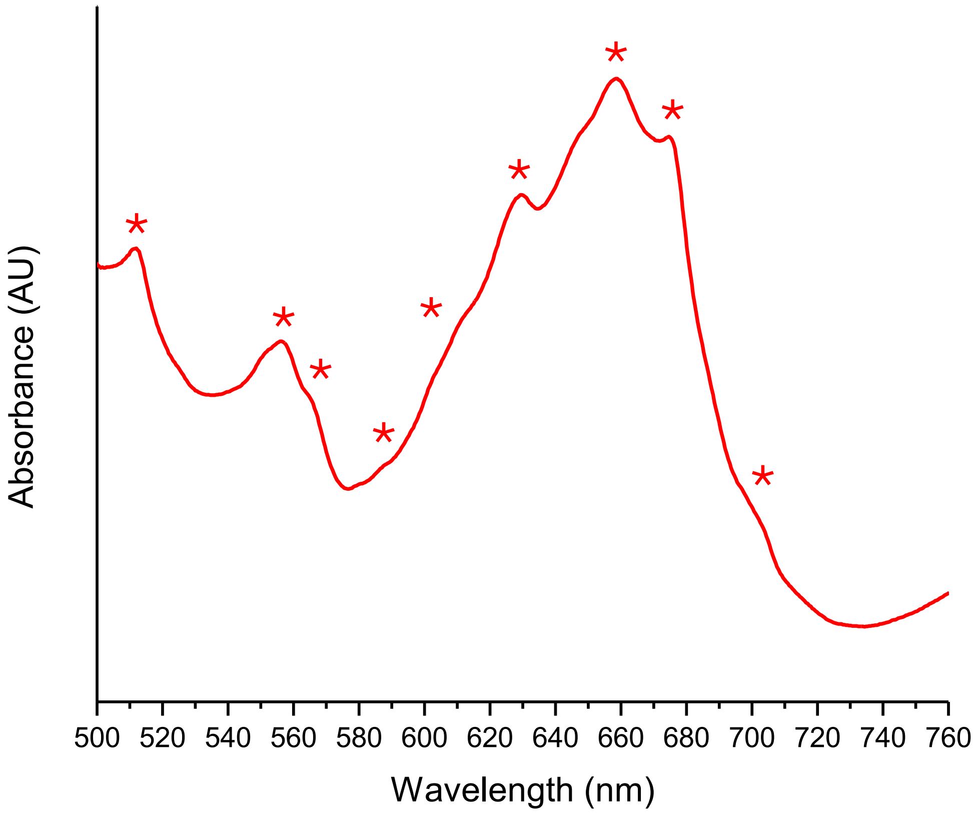

Figure S24. UV-Vis spectrum of **1** plotted from 500-750 nm. * = 512, 555, 565, 586, 610, 629, 658, 674, and 700 nm.

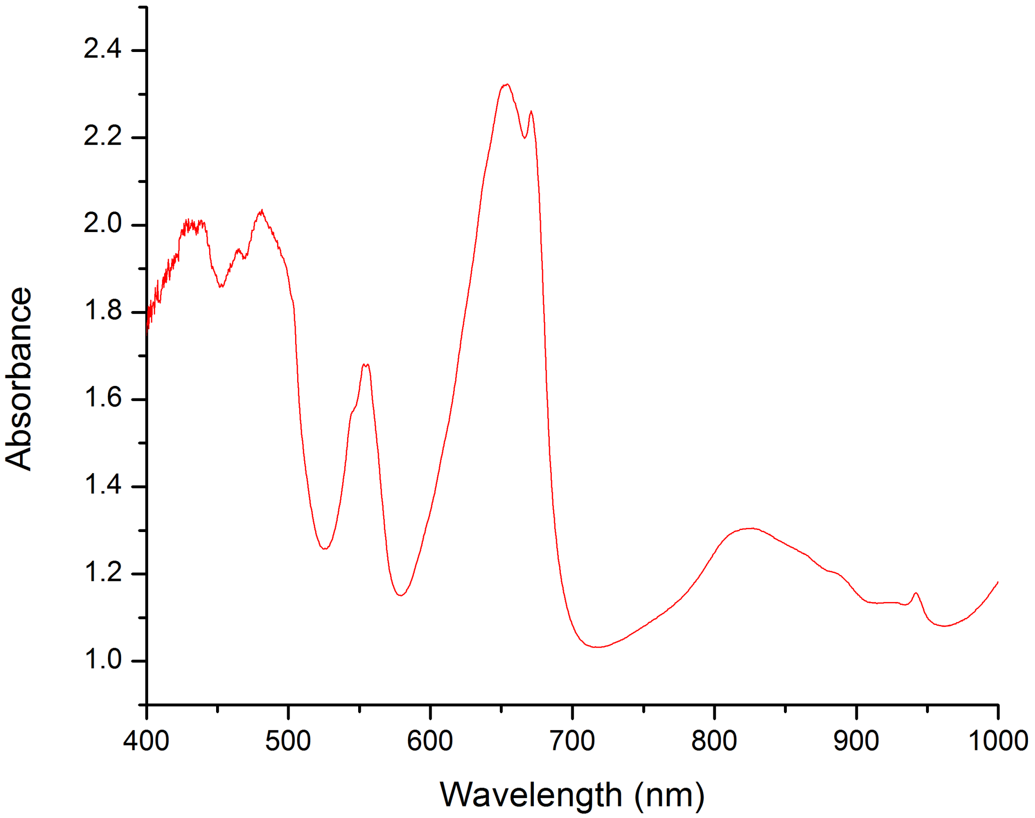

Figure S25. UV-Vis absorption spectrum of **2**. Bands are observed at 460, 480, 502, 545, 552, 556, 653, 670, 810, 950.

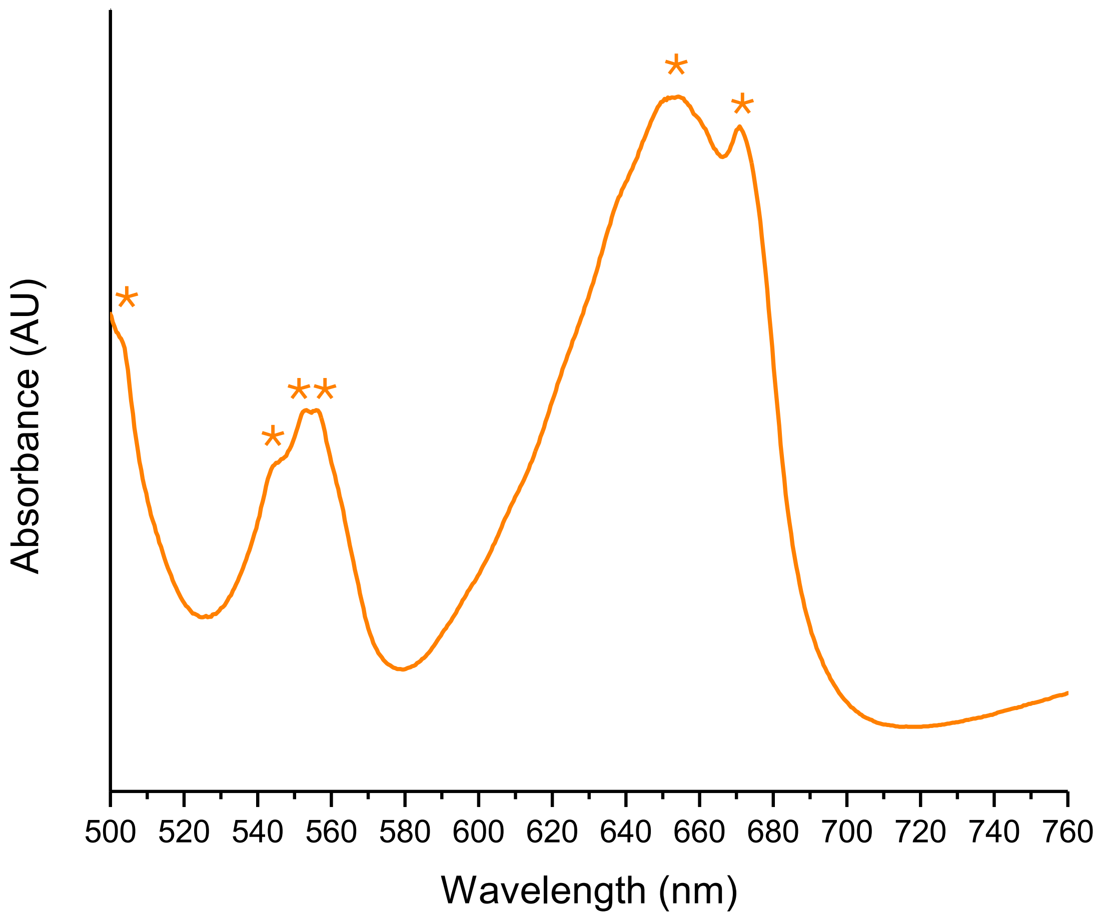

Figure S26. UV-Vis spectrum of **2** plotted from 500-750 nm. * = 502, 545, 552, 556, 653, and 670 nm.

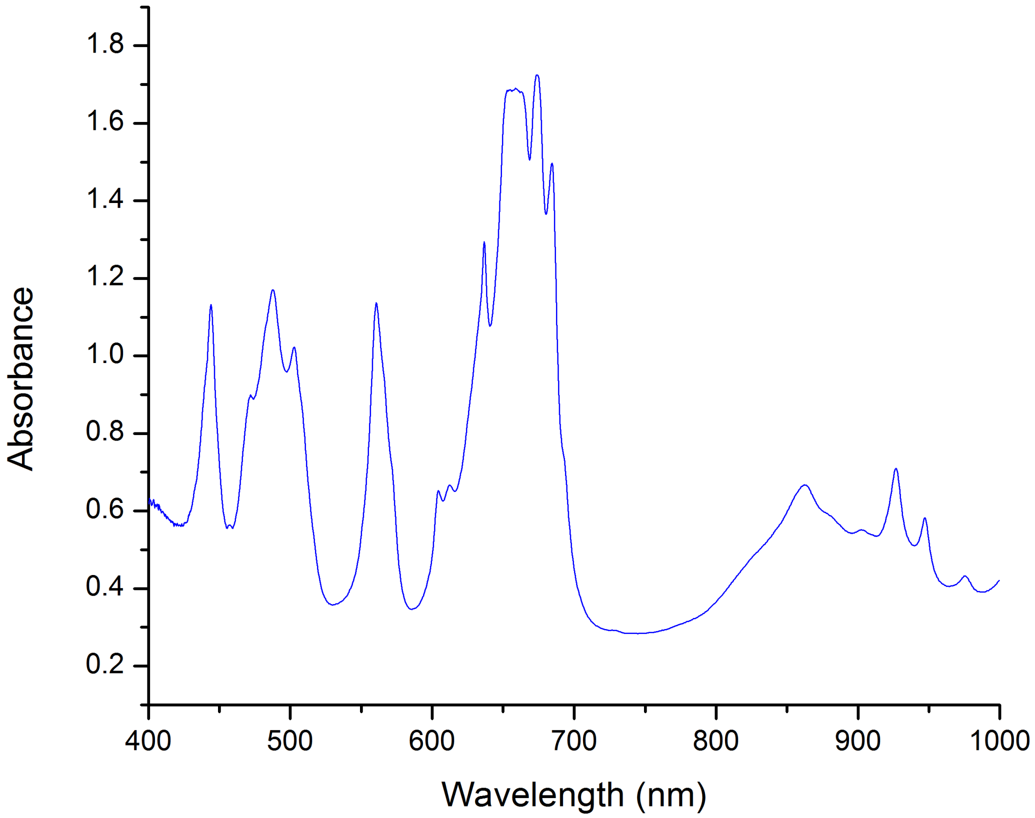

Figure S27. UV-Vis spectra of (**3**). Bands are observed at 450, 495, 502, 507, 560, 571, 603, 612, 636, 652, 658, 663, 673, 684, 692, 860, 900, 925, 950, 980.

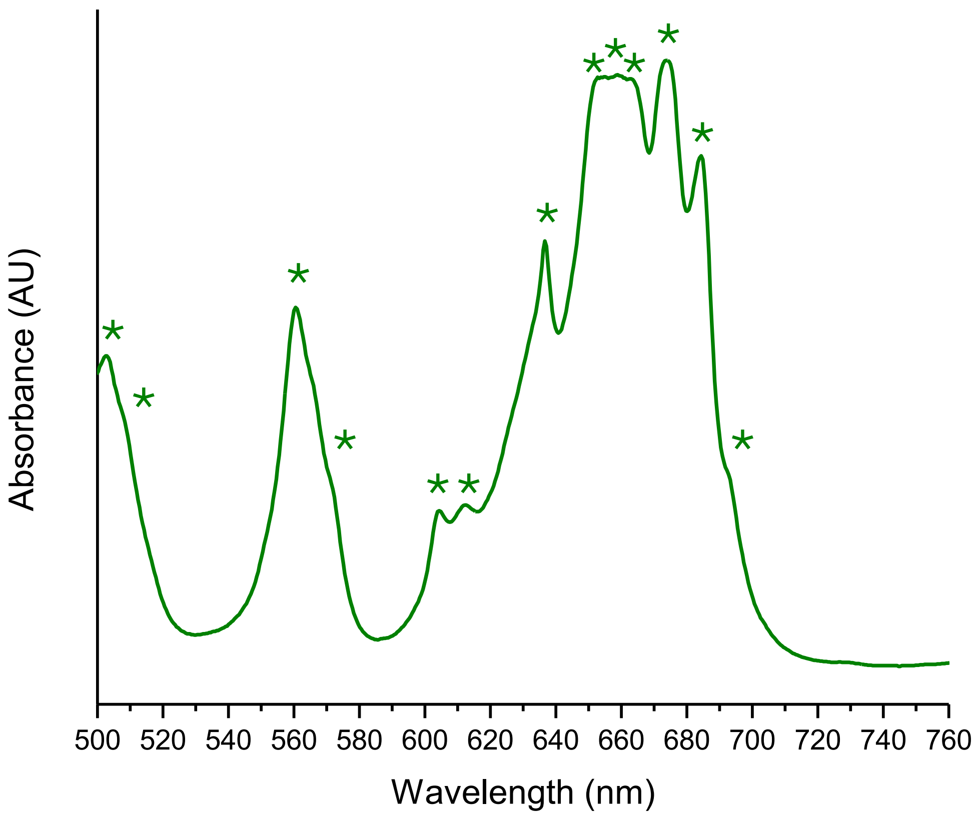

Figure S28. UV-Vis spectrum of **3** plotted from 500-750 nm. * = 502, 507, 560, 571, 603, 612, 636, 652, 658, 663, 673, 684, and 692 nm.

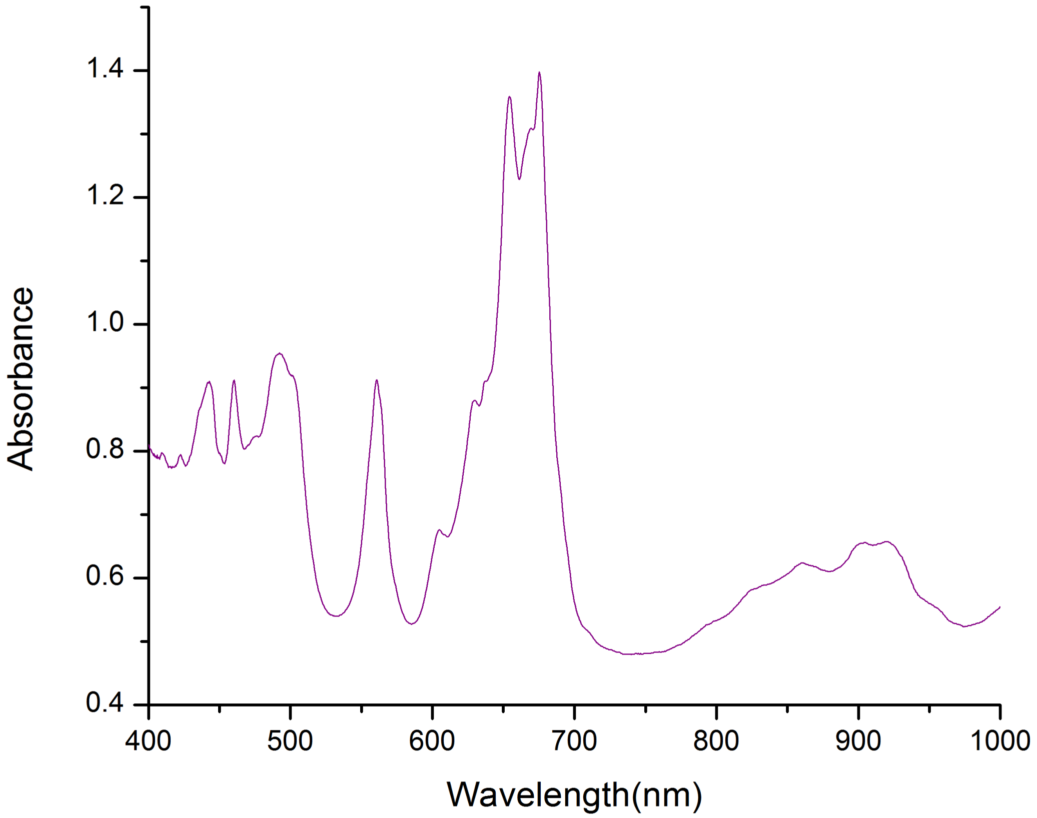

Figure S29. UV-Vis-NIR spectra of (**4**). Bands are observed at 445, 455, 495, 502, 561, 564, 605, 629, 636, 654, 669, 675, 855, 900, 990.

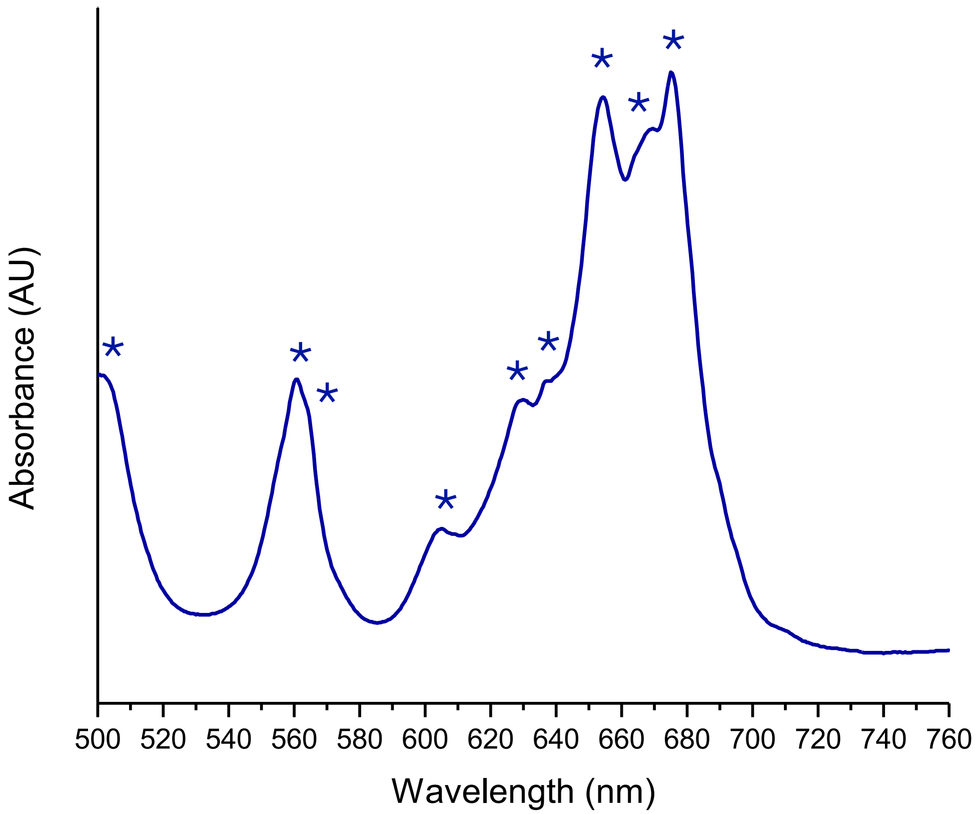

Figure S30. UV-Vis spectrum of **4** plotted from 500-750 nm. * = 502, 561, 564, 605, 629, 636, 654, 669, and 675 nm.

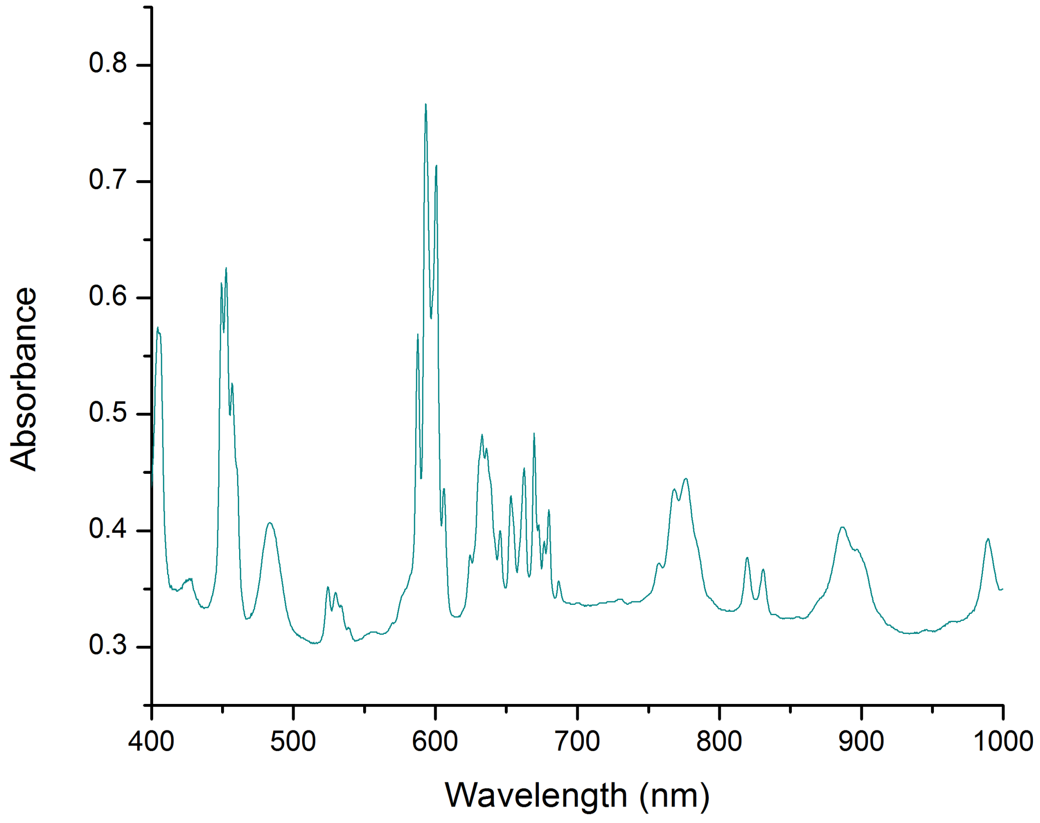

Figure S31. UV-Vis-NIR spectra of (**5**). Bands are observed at 402, 435, 450, 480, 524, 529, 533, 539, 569, 576, 587, 593, 600, 605, 623, 632, 636, 645, 653, 662, 669, 672, 676, 679, 686, 757, 770, 775, 820, 830, 885, 900, 990.

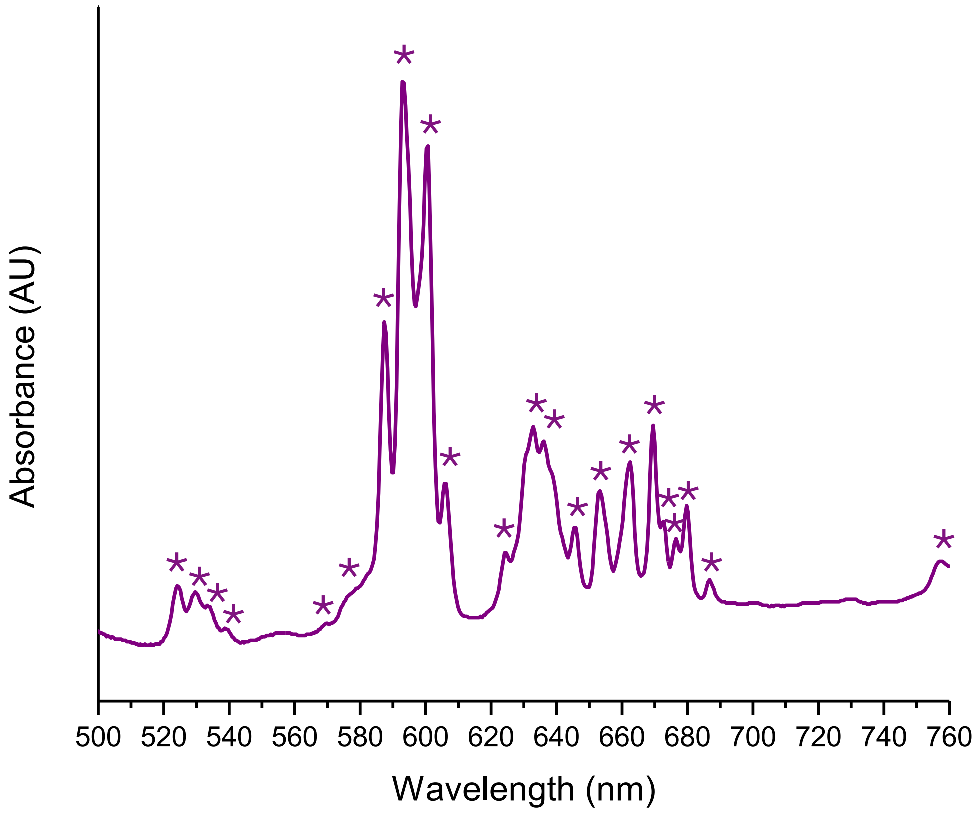

Figure S32. UV-Vis spectrum of **5** plotted from 500-750 nm. * = 524, 529, 533, 539, 569, 576, 587, 593, 600, 605, 623, 632, 636, 645, 653, 662, 669, 672, 676, 679, 686, and 757 nm.

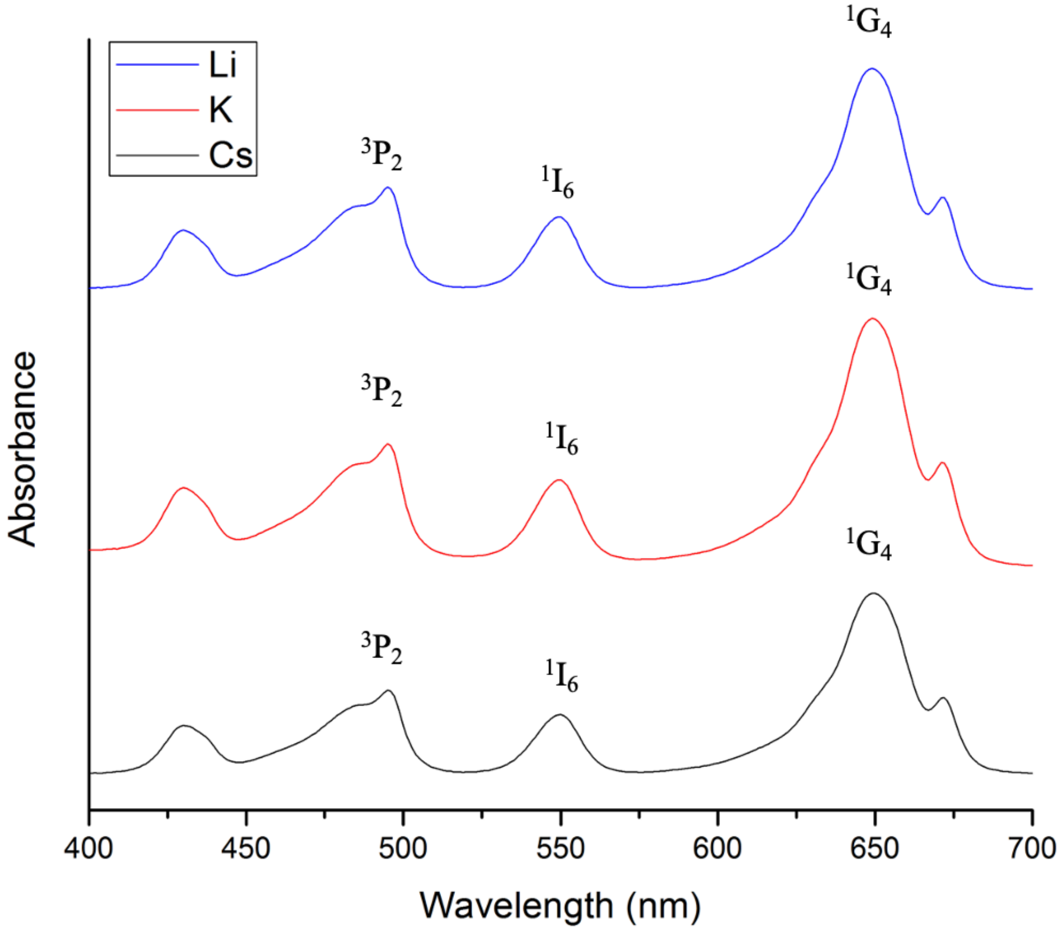

Figure S33. Solution-state UV-Vis spectra of the reactions solutions from which compounds **1** (blue), **3** (red), and **5** (black) were isolated. The spectra were collected after 1 day, prior to precipitation. The solution spectra are consistent with monomeric U-H_2O-Cl complexes.[2]

9. SUPRAMOLECULAR INTERACTIONS OBSERVED IN COMPOUNDS **1**–**3**.

Supramolecular interactions were calculated using the "Calc All" function within the PLATON software suite.[3] Significant noncovalent interactions for reported compounds – such as hydrogen bonding – are tabulated below, guided by the classification of supramolecular interactions as reported previously.[4,5] The relevant strengths of non-covalent interactions can be accessed by their interaction distance and angle. Hydrogen bonding can be defined as a D-H---A interaction, where D (donor) and A (acceptor) are typically atoms like oxygen or nitrogen. Strong hydrogen bonds are characterized by bond distances of 2.2-2.5 Å and bond angles of 170–180°, while moderate hydrogen bonds have distances ranging from 2.5-3.2 Å and angles greater than 130°.[4] Additionally, halogen bonding (D-H---A, where A = F, Cl, Br, or I), a subset of hydrogen bonding, typically features slightly longer bond distances. Strong halogen interactions are generally observed within the range of 3.0–3.6 Å.[5] Brief descriptions of the supramolecular networks are provided herein, and full summaries of the hydrogen bonding interactions, and outer coordination sphere A-X (A = Li, K, Rb; X = Cl, O) distances for each compound are provided in Tables S2-84.

Noncovalent Interaction Notations:

- D = donor atom involved in supramolecular interaction.
- A = acceptor atom involved in supramolecular interaction.

Table S2. Selected noncovalent hydrogen bonding interactions– observed in (**1**).

Interaction	Distance (Å), D–H---A	Angle (º), ∠D–H---A
O(6)-H --- (O5)	2.875(11)	100(6)
O(2)-H --- (Cl5)	3.228(5)	138(7)
O(5)-H --- (Cl5)	3.269(5)	165(7)
O(6)-H --- (Cl1)	3.329(4)	136(7)
O(6)-H --- (Cl2)	3.308(7)	145(8)

Table S3. Selected noncovalent hydrogen bonding interactions– observed in (**2**).

Interaction	Distance (Å), D–H---A	Angle (º), ∠D–H---A
O(1)-H --- (Cl1)	3.133(6)	146(9)
O(1)-H --- (Cl2)	3.068(7)	174(10)
O(2)-H --- (Cl1)	3.270(8)	163(9)

O(2)-H --- (Cl2)	3.110(6)	154(12)

Table S4. Selected noncovalent hydrogen bonding interactions– observed in (**3**).

Interaction	Distance (Å), D–H---A	Angle (º), ∠D–H---A
O(1)-H --- (Cl3)	3.149(3)	167(3)
O(1)-H --- (Cl5)	3.167(3)	156(3)
O(2)-H --- (Cl4)	3.286(3)	163(3)
O(2)-H --- (Cl5)	3.096(3)	165(4)
O(3)-H --- (Cl2)	3.361(3)	160(3)
O(3)-H --- (Cl5)	3.129(2)	130(3)
O(4)-H --- (Cl5)	3.061(3)	163(3)
O(4)-H --- (Cl5)	3.048(3)	164(4)

10. BOND DISTANCES OF COMPOUNDS **1-5**

Table S5. U-L (L = Cl, H_2O, μ_3O) distances for compounds **1-5**.

Compound	**1**	**2**	**3**	**4**	**5**
U – Cl (Å)	2.67 - 2.90	2.68 - 2.69	2.67 - 2.71	2.61 - 2.62	2.61
U – H_2O (Å)	2.50	2.42 - 2.44	2.38 - 2.46		
U – μ_3O (Å)	2.17 – 2.27				

Table S6. Li---L distances (L = Cl, O) for compound **1**.

Li---O/Cl Distances (Å)	Li 1	Li 2
Cl 2	2.5853 (213)	2.7839(18)
Cl 5	2.8512 (237)	
Cl 6	2.6989 (272)	
Average Li - Cl	2.712	2.784
O3	2.0928 (247), 2.0973 (214)	
O4	1.9156 (273)	
O5		1.9076 (68)
Average Li - O	2.035	1.9076

Table S7. K---Cl distances for compound **3**.

K---Cl Distances (Å)	K 1
Cl 1	3.0520 (12)

Cl 2	3.1267 (21), 3.1292 (13)
Cl 3	3.1176 (14)
Cl 4	3.1609 (12)
Cl 5	3.1326 (17)
Average	3.120

Table S8. Rb---Cl distances for compound **4**.

Rb---Cl Distances (Å)	Rb 1	Rb 2
Cl 1	3.4028 (80)	3.5877(96), 3.6352(92)
Cl 2	3.4977(91), 3.6259 (92)	3.3760(81)
Cl 3	3.3970 (80)	3.5918(93), 3.6390(96)
Cl 4	3.5131(93), 3.6162(93)	3.3829(79)
Cl 5	3.4035 (70)	3.5978(77), 3.6330(76)
Cl 6	3.5023(72), 3.6193(73)	3.3728 (80)
Average	3.509	3.535

Table S9. Summary of compounds 1-5.

Compound	Cation	Incorporation	Nuclearity	Symmetry	Supramolecular	Isomorphic phases
1	Li	Yes	4	C_s	3D	Yes
2	Na	No	1	C_2	3D	Yes
3	K	Yes	1	C_2	3D	Yes
4	Rb	Yes	1	C_3	0D	Yes
5	Cs	Yes	1	O_h	0D	No

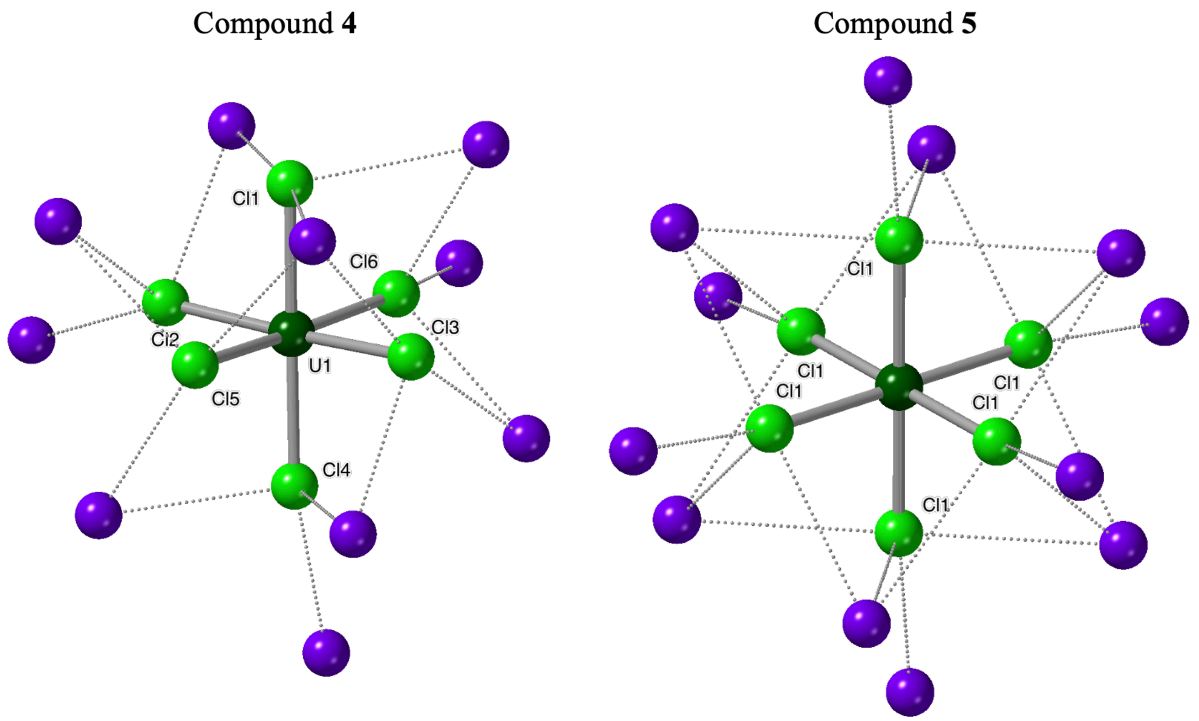

Figure S34. Diagram showing nearest cation interactions of **4** and **5**. Compound **4** shows 10 Rb---Cl interactions ranging from 3.3728-3.6390Å, while **5** displays 12 nearby Cs---Cl interactions ranging from 3.6613-3.7679Å.

11. COMPUTATIONAL DETAILS of 4 and 5

To confirm the origin of the experimentally observed differences between the measured optical spectra of $(Rb)_2[U(IV)(Cl)_6]$ (**4**) and $(Cs)_2[U(IV)(Cl)_6]$ (**5**), computations using multireference wave function-based methods were performed. First, state-average complete active space self-consistent field (SA-CASSCF) calculations were performed on the experimental crystal structure of $[U(IV)(Cl)_6]^{2-}$ including the rubidium and cesium counterions.[6] The active space included the seven 5f orbitals and corresponding two electrons, denoted (2e,7o). SA-CASSCF computations allows for the computation of 21 triplet and 28 singlet scalar-relativistic states arising from f–f excitations. To account for dynamic electron correlation, the SA-CASSCF energy levels were subjected to extended multistate second-order perturbation theory (XMS-CASPT2) calculations.[6–8] In the XMS-CASPT2 calculations, the zero-order Hamiltonian included an IPEA shift of 0.25 and an imaginary shift of 0.2 au, except for singlet states of $(Cs)_2[U(IV)(Cl)_6]$ where 0.3 au was required to eliminate intruder states.[9,10] Scalar-relativistic effects were included by employing Douglas–Kroll–Hess (DKH) Hamiltonian together with the relativistic ANO-RCC basis sets.[11] Specifically, the following contractions were used: [9s,8qp,6d,4f,2g,1h] for uranium, [5s,4p,2d,1f] for chlorine, [7s,6p,3d,1f] for rubidium, and [8s,7p,4d,2f,1g] for cesium. Cholesky decomposition along with local exchange screening were employed to reduce the cost of integral evaluation.[12] No symmetry was imposed in any of the calculations. All wave function-based computations were performed as implemented in the OpenMolcas program package.[13] The input and outputs of all performed calculations are provided in a long-term FigShare repository (DOI:10.6084/m9.figshare.28838264).

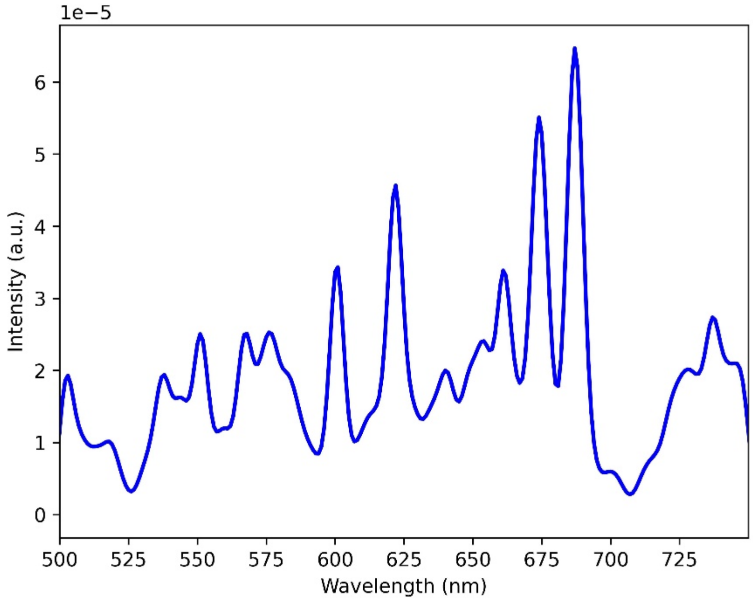

Figure S35. Calculated f-f UV-Vis spectra for $(Rb)_2[U(IV)(Cl)_6]$ extracted from the crystal structure of compound **4** at SO-XMS-CASPT2 level of theory. A Gaussian broadening of 5 nm was used to mimic the intrinsic experimental signal broadening.

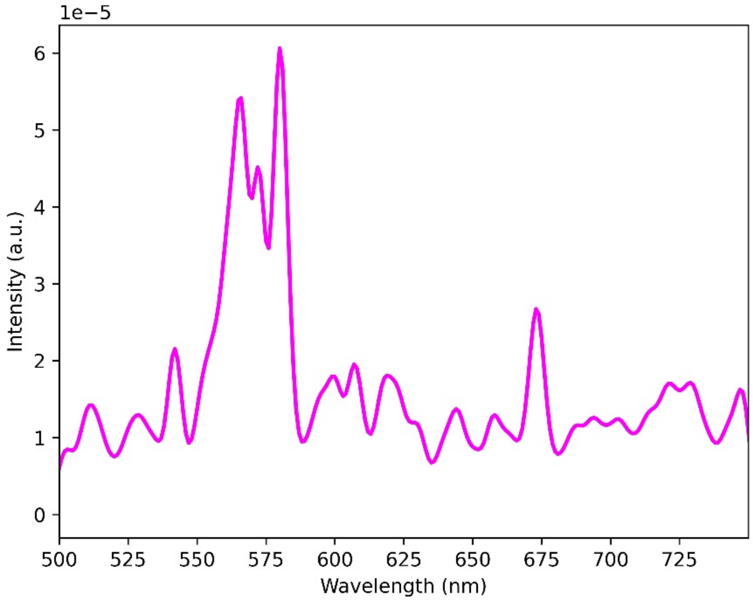

Figure S36. Calculated f-f UV-Vis spectra for $(Cs)_2[U(IV)(Cl)_6]$ extracted from the crystal structure of compound **5** at SO-XMS-CASPT2 level of theory. A Gaussian broadening of 5 nm was used to mimic intrinsic the experimental signal broadening.

Table S10. Cartesian coordinates used in the $(Rb)_2[U(IV)(Cl)_6]$ SO-XMS-CASPT2 calculation.

Cl	4.487909000000	9.881528000000	4.380234000000
Cl	4.917318000000	7.734878000000	7.273366000000
Cl	0.853740000000	9.343259000000	4.384867000000
Cl	1.055709000000	7.167062000000	7.261784000000
Cl	3.124637000000	6.468213000000	4.377918000000
Cl	2.485660000000	10.801337000000	7.266417000000
Rb	6.526984000000	6.424535000000	4.300320000000
Rb	6.523674000000	10.703704000000	7.217773000000
U	2.820741000000	8.564505000000	5.923392000000

Table S11. Cartesian coordinates used in the $(Cs)_2[U(IV)(Cl)_6]$ SO-XMS-CASPT2 calculation.

Cs	3.724800000000	2.150514000000	4.391302000000
Cl	-1.895178000000	-1.094182000000	-1.428669000000
Cl	0.000000000000	-2.188363000000	1.428669000000
Cl	-1.895178000000	1.094182000000	1.428669000000
Cl	1.895178000000	-1.094182000000	-1.428669000000
Cl	0.000000000000	2.188363000000	-1.428669000000
Cl	1.895178000000	1.094182000000	1.428669000000
Cs	0.000000000000	4.301029000000	1.591398000000
U	0.000000000000	0.000000000000	0.000000000000

12. REFERENCES.

(1) Sigmon, G. E.; Hixon, A. E. Eextension of the Plutonium Oxide Nanocluster Family to Include {Pu_{16}} and {Pu_{22}}. *Chem. – A Eur. J.* **2019**, *25* (10), 2463–2466. https://doi.org/https://doi.org/10.1002/chem.201805605.

(2) Wacker, J. N.; Vasiliu, M.; Huang, K.; Baumbach, R. E.; Bertke, J. A.; Dixon, D. A.; Knope, K. E. Uranium(IV) Chloride Complexes: UCl_6^{2-} and an Unprecedented $U(H_2O)_4Cl_4$ Structural Unit. *Inorg. Chem.* **2017**, *56* (16), 9772–9780. https://doi.org/10.1021/acs.inorgchem.7b01293.

(3) Spek, A. L. Single-Crystal Structure Validation with the Program PLATON. *J. Appl. Crystallogr.* **2003**, *36* (1), 7–13. https://doi.org/10.1107/S0021889802022112.

(4) Thomas, S. The Hydrogen Bond in the Solid State. *Angew. Chemie Int. Ed.* **2002**, *41*, 48–76.

(5) Cavallo, G.; Metrangolo, P.; Milani, R.; Pilati, T.; Priimagi, A.; Resnati, G.; Terraneo, G. The Halogen Bond. *Chem. Rev.* **2016**, *116* (4), 2478–2601. https://doi.org/10.1021/acs.chemrev.5b00484.

(6) Roos, B. O.; Taylor, P. R.; Sigbahn, P. E. M. A Complete Active Space SCF Method (CASSCF) Using a Density Matrix Formulated Super-CI Approach. *Chem. Phys.* **1980**, *48* (2), 157–173. https://doi.org/https://doi.org/10.1016/0301-0104(80)80045-0.

(7) Granovsky, A. A. Extended Multi-Configuration Quasi-Degenerate Perturbation Theory: The New Approach to Multi-State Multi-Reference Perturbation Theory. *J. Chem. Phys.* **2011**, *134* (21), 214113. https://doi.org/10.1063/1.3596699.

(8) Andersson, K.; Malmqvist, P. A.; Roos, B. O.; Sadlej, A. J.; Wolinski, K. Second-Order Perturbation Theory with a CASSCF Reference Function. *J. Phys. Chem.* **1990**, *94* (14),

5483–5488. https://doi.org/10.1021/j100377a012.
(9) Forsberg, N.; Malmqvist, P.-Å. Multiconfiguration Perturbation Theory with Imaginary Level Shift. *Chem. Phys. Lett.* **1997**, *274* (1), 196–204. https://doi.org/https://doi.org/10.1016/S0009-2614(97)00669-6.
(10) Ghigo, G.; Roos, B. O.; Malmqvist, P.-Å. A Modified Definition of the Zeroth-Order Hamiltonian in Multiconfigurational Perturbation Theory (CASPT2). *Chem. Phys. Lett.* **2004**, *396* (1), 142–149. https://doi.org/https://doi.org/10.1016/j.cplett.2004.08.032.
(11) Roos, B. O.; Lindh, R.; Malmqvist, P.-Å.; Veryazov, V.; Widmark, P.-O.; Borin, A. C. New Relativistic Atomic Natural Orbital Basis Sets for Lanthanide Atoms with Applications to the Ce Diatom and LuF3. *J. Phys. Chem. A* **2008**, *112* (45), 11431–11435. https://doi.org/10.1021/jp803213j.
(12) Aquilante, F.; Malmqvist, P.-Å.; Pedersen, T. B.; Ghosh, A.; Roos, B. O. Cholesky Decomposition-Based Multiconfiguration Second-Order Perturbation Theory (CD-CASPT2): Application to the Spin-State Energetics of CoIII(Diiminato)(NPh). *J. Chem. Theory Comput.* **2008**, *4* (5), 694–702. https://doi.org/10.1021/ct700263h.
(13) Fdez. Galván, I.; Vacher, M.; Alavi, A.; Angeli, C.; Aquilante, F.; Autschbach, J.; Bao, J. J.; Bokarev, S. I.; Bogdanov, N. A.; Carlson, R. K.; et al. OpenMolcas: From Source Code to Insight. *J. Chem. Theory Comput.* **2019**, *15* (11), 5925–5964. https://doi.org/10.1021/acs.jctc.9b00532.
